# 3DSC - a dataset of superconductors including crystal structures

**DOI:** 10.1038/s41597-023-02721-y

**Published:** 2023-11-21

**Authors:** Timo Sommer, Roland Willa, Jörg Schmalian, Pascal Friederich

**Affiliations:** 1 Institute of Theoretical Informatics, Karlsruhe Institute of Technology, Engler-Bunte-Ring 8, 76131 Karlsruhe, Germany; 2 Institute for Theory of Condensed Matter, Karlsruhe Institute of Technology, Wolfgang-Gaede-Str. 1, 76131 Karlsruhe, Germany; 3 School of Chemistry, Trinity College Dublin, College Green, Dublin, 2 Ireland; 4 Institute for Quantum Materials and Technologies, Karlsruhe Institute of Technology, Hermann-von-Helmholtz-Platz 1, 76344 Eggenstein-Leopoldshafen, Germany; 5 Institute of Nanotechnology, Karlsruhe Institute of Technology, Hermann-von-Helmholtz-Platz 1, 76344 Eggenstein-Leopoldshafen, Germany

**Keywords:** Superconducting properties and materials, Scientific data, Software

## Abstract

Data-driven methods, in particular machine learning, can help to speed up the discovery of new materials by finding hidden patterns in existing data and using them to identify promising candidate materials. In the case of superconductors, the use of data science tools is to date slowed down by a lack of accessible data. In this work, we present a new and publicly available superconductivity dataset (‘3DSC’), featuring the critical temperature *T*_*C*_ of superconducting materials additionally to tested non-superconductors. In contrast to existing databases such as the SuperCon database which contains information on the chemical composition, the 3DSC is augmented by approximate three-dimensional crystal structures. We perform a statistical analysis and machine learning experiments to show that access to this structural information improves the prediction of the critical temperature *T*_*C*_ of materials. Furthermore, we provide ideas and directions for further research to improve the 3DSC. We are confident that this database will be useful in applying state-of-the-art machine learning methods to eventually find new superconductors.

## Background & Summary

Superconductors are materials in which the electrical resistance is zero when the temperature drops below a critical temperature *T*_*c*_. Furthermore, superconductors are perfect diamagnets that expel magnetic fields via the Meissner effect. These properties make superconductors very useful for many high-power applications such as efficient electric power conversion, lossless power transmission, and ultra-strong magnets, as well as high-sensitivity sensor materials e.g. superconducting quantum interference devices and photon detectors^1,2^. The discovery of new superconducting materials with optimized properties will enable e.g. the use of cheaper coolants due to increased critical temperatures, stronger magnets due to improved magnetic properties, and simpler production of superconducting wires due to improved mechanical properties.

The critical temperature *T*_*c*_ can be very sensitive to small changes in the crystal structure, for example to changes in the interatomic distances via mechanical pressure or chemical pressure^3^, i.e. the deformation of the lattice by replacing one atom with another element with same valency but different size. Despite the success of understanding the mechanism behind superconductivity within a microscopic theory, such as the theory by Bardeen, Cooper and Schrieffer^4^ and strong-coupling generalizations thereof, it is to date difficult to faithfully predict the critical temperature *T*_*c*_ of new materials. Even though the prediction of the critical temperature has improved a lot over the last years and decades for well-understood classes of superconductors, the predictability of the superconducting onset temperature remains a major challenge in the material’s research endeavour, in particular in view of exploring new material candidates. This is largely caused by the dependence of *T*_*c*_ on subtle details of atomic arrangements in the crystal structure. Input parameters of the microscopic, low-energy theories, such as the electronic density of states at the Fermi level, the phonon spectrum, and the electron-lattice coupling, are not easily related to the chemical formula. Thus, when predicting the critical temperature *T*_*c*_ with machine learning, a first step to narrow this gap is to have access not only to the chemical composition of the material, but also to the exact 3D structure of the crystal.

Machine learning has been widely used for the prediction of materials properties. Saal *et al*.^5^ collected and reviewed a big number of machine learning generated predictions which have been confirmed experimentally afterward in applications ranging from organic LEDs over new binary and ternary crystal structures, perovskites, metallic glasses and metal-organic-frameworks to superhard materials. Furthermore, machine learning was used to predict the critical temperature *T*_*c*_ of superconductors using the SuperCon database^6^ as training data, which is the largest and most commonly used dataset of superconductors. Previous papers have also made preprocessed subsets of the data available for further research^7,8^.

There have been many attempts to predict the critical temperature *T*_*c*_ of a material using the SuperCon database. Hamidieh^8^ used a gradient boosting model (XGB^9^) trained on MAGPIE features^10^ to predict *T*_*c*_. Aketi *et al*.^11^ used gradient-boosted decision trees, Matsumoto *et al*.^12^ used random forests and Le *et al*.^13^ used Bayesian neural networks on very similar features, while Gaikwad *et al*.^14^ compare multiple machine learning models. Konno *et al*.^15^ and Zeng *et al*.^16^ used a convolutional neural network (CNN) and represented the chemical formula as elements on a grid. Li *et al*.^17^ used a hybrid neural network consisting of a CNN and a recurrent neural network (RNN) which is trained on Atom2Vec features^18^. Dan *et al*.^19^ use a convolutional gradient boosting decision tree (ConvGBDT). Sizochenko *et al*.^20^ found that an often-used subset of the SuperCon contained a lot of duplicate entries and repeated their analysis with the cleaned dataset. Meredig *et al*.^21^ showed that random splits for the cross-validation give overly confident model evaluations. Roter *et al*.^22^ trained a bagged tree model on the chemical composition and argued that physical features such as the Fermi energy would be helpful for increasing the performance of their model if they were available for more materials.

Data availability is the most important prerequisite for the development of (supervised) machine learning models for materials property prediction. In particular, informative and complete information on the materials is essential for the training of accurate machine learning models. All of the studies discussed above were based on representing materials only by their chemical composition, which is not a unique and complete representation of materials. Yet, most SuperCon entries contain only the chemical formula and critical temperature *T*_*c*_ of each material. Structural data such as space group and crystal system are only sparsely recorded and the full three-dimensional crystal structure is never given. Therefore, all of the aforementioned predictions of the critical temperature using the SuperCon database were limited to representations of the chemical composition of each material.

One notable exception of using only chemical formulas to predict critical temperatures is the work of Stanev *et al*.^7^. They developed a superconductivity classifier based on matching the chemical compositions of materials in the SuperCon with the chemical compositions of materials in the AFLOW database and used tabular structural and electronic features such as the space group and the energy per atom as additional features. In this pioneering work, 1500 materials could be matched, half of them being superconductors. Stanev *et al*. argued that structural information is helpful in predicting superconductivity, yet realized the issue of severely reducing the size of the dataset when doing this matching. As of today, the matched crystal structures were not published.

Recently, two more databases dealing with superconductors were presented in the literature. The SuperMat^23^ database and the SC-CoMIcs^24^ database are corpora of manually annotated texts from papers about superconductors. The annotations consist of different entities such as chemical formula and critical temperature with which certain phrases in the texts have been labeled. These corpora can be used for tasks such as training a named entity recognition model such as SciBERT^25^ on automatically labeling new papers, which was demonstrated by Yamaguchi *et al*.^24^ Foppiano *et al*.^23^ also publicly provide their annotation procedure to encourage others to continue this work. So far, these annotated corpora are not publicly available. In the future, they might be useful to automatically extract information about superconductivity from literature.

Court *et al*.^26^ used the already trained ChemDataExtractor^27^ to extract information of superconductors and magnetic materials from literature. They found approximately 20,400 superconductors and magnetic materials together with their chemical compositions and respective phase transition temperatures. The focus of the study was the prediction of the phase diagram of magnetic and superconducting materials. Furthermore, some of the entries were paired with crystal structures from the Crystallographic Open Database (COD)^28^. The authors provide a link to an interactive web app and the data, yet, the provided link is currently inactive. Another recently initiated superconductor database is the Superconducting Research Database^29^. In this online database, superconductors can be submitted with their exact three-dimensional crystal structure and critical temperature *T*_*c*_. This database currently contains 14 superconductors which limits its usefulness for machine learning processes.

In this work, we extended the structure matching approach by Stanev *et al*.^7^ to build a new database (called 3DSC) of experimentally tested superconducting and non-superconducting materials^30^. This database is made publicly available. The 3DSC database features the critical temperature of superconductors as well as the approximated 3D crystal structure of each material. The core idea is to match materials in the SuperCon database with (modified) crystal structures of the Materials Project^31,32^ and the Inorganic Crystal Structure Database^33–35^ (ICSD). In addition to matching only exact chemical compositions (as in Stanev *et al*.^7^), we employ a systematic adaptation algorithm that approximates the three-dimensional crystal structures of materials without perfect match by artificial doping of similar crystal structures. For example, the crystal structure of the SuperCon entry CuLa_1.95_Nd_0.05_O_4_ (which has no perfect match in the Materials Project database) is approximated by taking the 3D crystal structure of CuLa_2_O_4_ and partially replacing La with Nd at the respective crystal positions. This step is important to maximize the number of matched materials since the SuperCon contains many entries with doped materials, which otherwise would mostly be discarded.

In this paper, we introduce and analyze two different 3DSC databases. Both are based on the SuperCon database, but one uses structures from the Materials Project (3DSC_MP_) and one uses structures from the ICSD (3DSC_ICSD_). Using our matching and adaptation algorithm, we are able to match 5,759 (3DSC_MP_) and 9,150 (3DSC_ICSD_) superconducting and non-superconducting materials from the SuperCon. We publicly provide the full 3DSC_MP_ dataset on figshare^30^ including the critical temperature *T*_*c*_ and approximate three-dimensional crystal structures. However, structures from the ICSD must not be (re-)published. Therefore, we refrain from publishing the 3DSC_ICSD_. The subset of the 3DSC_ICSD_ that we provide under this link only contains the ICSD IDs necessary for reproducing the full dataset. The necessary structures can be downloaded with an ICSD license and artificially doped using our code in the aforementioned repository. However, despite not being able to publish this database, we have decided to present the 3DSC_ICSD_ in this paper along with the 3DSC_MP_, since it contains more structures and slightly different information than the 3DSC_MP_.

## Methods

### Overview of 3DSC data generation

**Fig. 1:**
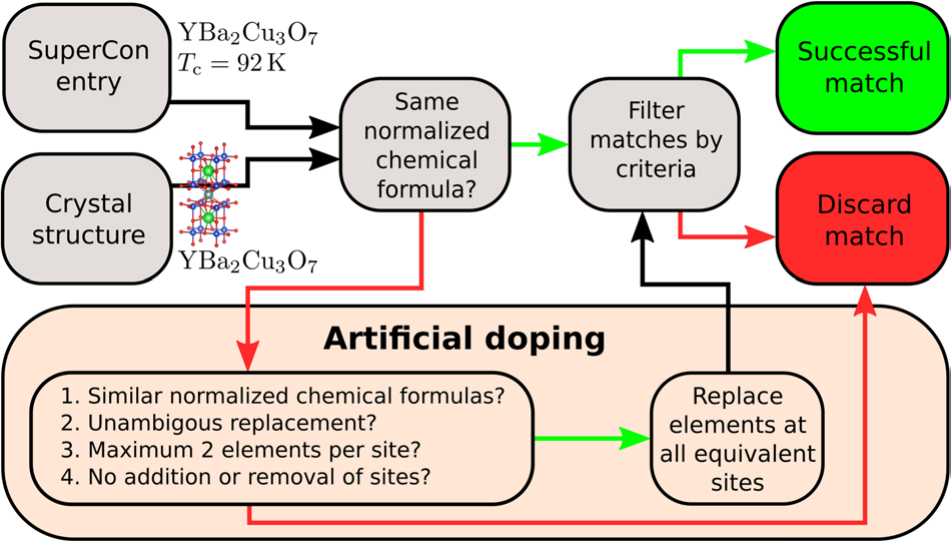
A schematic view of the matching and adaptation algorithm.

In this section, we describe our algorithm to match entries of the SuperCon database based on their chemical formula with 3D structures from crystal structure databases (see Fig. 1). We use and compare two different crystal structure databases, the Materials Project and the ICSD. We furthermore use the copy of the SuperCon database published by Stanev *et al*.^7^. All databases are cleaned as described in Sec. ‘Data and dataset cleaning’.

In the first step of the matching algorithm to build our 3DSC database, each SuperCon entry is paired with each crystal structure. If the chemical formula matches perfectly after normalization, the SuperCon entry is paired to the crystal structure and added to our database. If the chemical formula is close but not equal, we performed an artificial doping process to modify the crystal structure and to aim for a perfect match with the chemical formula of the SuperCon entry. This process is described in more detail in Sec. ‘Matching algorithm of SuperCon entries and 3D crystal structures’.

Because this matching and adaptation algorithm generally matches multiple crystal structures to each SuperCon entry, we conclude the process by filtering the matches according to specific criteria and keeping only the most optimal matches. After applying this filter, we are left with the final 3DSC database which contains chemical formula, critical temperature *T*_*c*_, and the (in many cases approximate) crystal structure. In the case of the Materials Project database, the final 3DSC_MP_ dataset contains 5,759 SuperCon entries matched with 5,773 crystal structures. In the case of the ICSD, the final 3DSC_ICSD_ dataset contains 9,150 SuperCon entries matched with 86,490 crystal structures. One reason for this high number of matched crystal structures is that the ICSD has a large number of entries with the same crystal structure at different crystal temperatures.

### Data and dataset cleaning

#### SuperCon

The SuperCon database is the largest database of superconductors and has been used multiple times in the literature to predict the critical temperature of superconductors with machine learning methods. It contains approximately 33,000 materials that have been tested for superconductivity. Approximately 10,000 of the entries are duplicates of the same material with the same chemical formula. However, the SuperCon database features only the chemical formula of each material, whereas structural data such as space group and lattice type is only sparsely recorded and the full crystal structure is never given. Since the SuperCon database itself was unavailable at the beginning of this work and has only recently been made available again, in this study we use the already cleaned and published subset of the SuperCon dataset published by Stanev *et al*.^7^ which contains 16,400 different materials with approximately 4,000 non-superconductors. The dataset can be found on GitHub (https://github.com/vstanev1/Supercon) and includes the chemical formula and the critical temperature *T*_c_ of each material.

We found that this dataset was not fully cleaned yet. We assume that in the original study chemical formulas were compared as strings, but sometimes the order of elements in the string was different even though it was the same material. For these 21 materials, we averaged the critical temperatures according to the same algorithm as used in Stanev *et al*., i.e. taking the mean and excluding the material if the standard deviation was greater than 5 K. Because we are averaging over data points some of which are already averaged, the resulting average might not be the same as in the original dataset. Additionally, we found that some chemical formulas were invalid, e.g. in the formula Bi_4.4_Sr_3.6_Ca_2_Cu_4_OY the Y was considered to represent yttrium, but it actually represented an unknown quantity of oxygen (the SuperCon database strictly follows a nomenclature, where each element has a count, even if the count is 1). Excluding these entries reduced the dataset by 128 entries. We also excluded 4 entries that had chemical formulas with more than 150 atoms because these are likely mistakes in the database. Finally, we decided to exclude the few entries with the heavy elements americium (Am), curium (Cm), and polonium (Po) to reduce the number of entries with rarely occurring elements. After all cleaning steps, we are left with 15,758 SuperCon entries of which 3,854 are non-superconductors. The maximum critical temperature is 143 K for Ba_2_Ca_1.98_Cu_2.9_Hg_0.66_Pb_0.34_O_8.4_. Non-superconductors are encoded as having a critical temperature of 0 K.

#### Crystal structure datasets

As sources for the crystal structures, we used the Materials Project and the ICSD. The Materials Project contains approximately 139,000 DFT calculated structures and electronic features and is openly accessible. The ICSD contains approximately 243,000 mostly experimental structures and is accessible only with a license. The ICSD database was cleaned before further processing: 19,077 DFT calculated (rather than experimentally measured) entries were excluded to make the dataset more consistent. 18,147 entries were excluded because the chemical composition given by the ICSD and extracted from the crystal structure using the python package pymatgen^36^ were inconsistent. Similarly, 3,195 entries were excluded because the space group given by the ICSD and the one recognized with pymatgen were inconsistent. For space group analysis we used an angle tolerance of 5° (the pymatgen default) and a symmetry tolerance of 0.1Å for the Materials Project structures (the value recommended in pymatgen for less well refined structures) and values of 0.01Å  (the pymatgen default) and 0.1Å  for the ICSD structures and excluded all crystal structures with unclear space groups. Finally, 3,132 more entries were excluded because of an invalid chemical formula. Even though not technically invalid, we decided to also exclude materials including deuterium and tritium. Because the ICSD also has the crystal temperature *T*_cry_ recorded for most materials, entries without crystal temperature were assumed to be recorded at room temperature (293 K).

The Materials Project was checked as well but no entries had to be excluded due to the aforementioned reasons. However, materials without recorded *E*_hull_ were excluded since this feature was needed for the matching and adaptation algorithm (see Sec. ‘Matching algorithm of SuperCon entries and 3D crystal structures’). Because the Materials Project structures had no crystal temperature given, the crystal temperature *T*_cry_ was set to 0 K in order to have a consistent set of features for the machine learning models.

### Matching algorithm of SuperCon entries and 3D crystal structures

The following section describes the algorithm used for matching and adaptation (from now on referred to as *artificial doping*) of SuperCon entries and crystal structures in more detail. The aim is to find one or multiple crystal structures for as many SuperCon entries as possible. Therefore, each SuperCon entry is paired with each crystal structure and the similarities between the chemical compositions are compared. In order to increase the number of matched entries, we normalize the chemical formulas before matching and perform artificial doping to approximate crystal structures of materials where the crystal structure of a very similar material is known.

#### Normalization of chemical formulas

Instead of matching chemical formulas only when they match exactly, we also match chemical formulas if they differ by a constant factor. For example, the SuperCon entry CuLa_2_O_4_ would be matched by a crystal structure with the chemical formula Cu_2_La_4_O_8_ with a relative factor of 1/2. This normalization procedure increases the matched entries by a large factor. We attribute this finding to potential experimental difficulties in determining the exact number of atoms in the unit cell of each material.

#### Artificial doping

If the chemical formulas of SuperCon entry and crystal structure do not match perfectly but are still similar, we perform artificial doping. Artificial doping means that we use a crystal structure with a similar chemical formula as a proxy crystal structure for the real crystal structure of the SuperCon entry. We then partially replace the atoms at given crystal positions with other chemical elements, imitating real physical doping. Statistically occurring vacancies can be introduced as well by using nothing as dopant and simply reducing the occupancy of the regarding crystal site. After the replacement, the chemical formula of the new crystal structure matches perfectly the required chemical formula of the SuperCon entry. Note that this algorithm only changes the occupancies of the crystal sites. It does not change coordinates or interatomic distances. Besides, this algorithm can only be applied if the original chemical formulas are close enough, so that the real crystal structure of the SuperCon entry is likely to have similar crystal parameters (such as space group and lattice parameters) as the proxy crystal structure. Therefore, in order to keep the introduced bias small, we perform artificial doping only if the following requirements are met:

- *The chemical formulas are similar:* We define three similarity metrics of chemical formulas, which are checked after normalizing the chemical formula of the crystal structure as explained above. These metrics are the absolute difference of atom numbers
  1 $${\Delta }_{{\rm{abs,i}}}=\left|{x}_{{\rm{sc,i}}}-{x}_{{\rm{cry,i}}}\right|,$$
  the relative difference of atom numbers
  2 $${\Delta }_{{\rm{rel,i}}}=\frac{2\left|{x}_{{\rm{sc,i}}}-{x}_{{\rm{cry,i}}}\right|}{{x}_{{\rm{sc,i}}}+{x}_{{\rm{cry,i}}}},$$
  and the total weighted relative difference
  3 $${\Delta }_{{\rm{totrel}}}=\frac{2{\sum }_{i}\left|{x}_{{\rm{sc,i}}}-{x}_{{\rm{cry,i}}}\right|}{{\sum }_{i}{x}_{{\rm{sc,i}}}+{x}_{{\rm{cry,i}}}}.$$
  *x*_sc,i_ and *x*_cry,i_ are the quantities of element *i* of the chemical formulas of SuperCon entry and crystal structure, respectively. A pair consisting of a SuperCon entry and a crystal structure is considered similar if ${\Delta }_{{\rm{a}}{\rm{b}}{\rm{s}},{\rm{i}}}\le 0.30{{\rm{O}}{\rm{R}}\Delta }_{{\rm{r}}{\rm{e}}{\rm{l}},{\rm{i}}}\le 0.20{\rm{\forall }}i$ and Δ_totrel_ ≤ 0.15, and the SuperCon entry has the same elements or up to one additional element as the crystal structure. One exception is that pure elements only match the same pure element.
  Since artificial doping makes the assumption that the structure of the initial and final crystal are close, we chose a SuperCon-crystal structure match to be acceptable, if the chemical formulas were similar enough so that the crystal structures of those materials would probably have the same space group. On the other hand, if the chemical formulas were different enough so that they would probably have different space groups, this match was considered to be not acceptable. Based on these considerations, we assigned a number of randomly selected example matches into acceptable and not acceptable and then chose the thresholds in a sensible way such that they would mirror the manual assignments. Note that the exact threshold values only partially influence the final result, as only the most optimal matches according to additional criteria (see later) will be added to the final datasets. Table 1 illustrates the procedure by showing examples of chemical formulas and whether they are considered similar, based on the definitions above.

**Table 1:** Examples of pairs of chemical formulas of SuperCon entries and crystal structures. For each pair, the columns show the three similarity metrics (Eqs. 1–3). The subjective criterion for deciding if a match should be accepted or not was based on the consideration if both materials would have the same space group or not.

SuperCon	Crystal structure	max(Δ_abs_)	max(Δ_rel_)	Δ_totrel_	Comment
CuLa_1.95_Nd_0.05_O_4_	CuLa_1.90_Nd_0.10_O_4_	0.05	0.67	0.01	Matches after modification of the stoichiometry of La and Nd.
CuLa_1.95_Nd_0.05_O_4_	CuLa_2_O_4_	0.05	2.0	0.01	Matching with modification. One site (La) of the unit cell is partially replaced by another element (Nd).
CuLa_2_O_4_	Cu_2_La_4_O_8_	0	0	0	Matches perfectly (normalized chemical formulas are identical).
NbGeC_1.5_N_0.5_	NbGeC_1.0_N_1.0_	0.5	0.67	0.25	Does not match because each metric is above its threshold (severe stoichiometry change would be required).
W_5_Tc_7_	W_6_Tc_6_	1	0.18	0.17	Does not match because Δ_totrel_ is above the threshold of 0.15.
  The upper bound on Δ_rel,i_ ensures that for each element, the relative difference of the chemical formulas is at most 20%. However, this requirement does not work well for doped materials such as CuLa_1.95_Nd_0.05_O_4_. This chemical formula is close to one with a higher Nd doping concentration (e.g. CuLa_1.90_Nd_0.10_O_4_), even though Δ_rel_ is 67% for Nd. Therefore, the metric Δ_abs,i_ allows for absolute differences of 0.3 or less for an element even though the requirement on Δ_rel_ would be violated. The metric Δ_totrel_ ensures that the 20% boundary is maxed out preferably for elements with a low number of atoms (and therefore low weight) in the chemical formula.
- *The* *necessary* *replacement of elements for artificial doping is unambiguous:* In a real crystal structure, not all crystal sites occupied by the same chemical element are equivalent. The dopant might prefer specific crystal sites due to differences in the local environment and thus free energy. Without further analysis, our artificial doping algorithm cannot determine which of the possible crystal sites becomes doped. Therefore, artificial doping is performed only if there is not more than one set of equivalent crystal sites for the dopants. In this case, the dopants are distributed equally over all equivalent crystal sites (see Fig. 2). In Fig. 2a, there is only one set of equivalent Rh sites. Therefore Rh can be unambiguously replaced with Ir. In Fig. 2b there are two sets of equivalent Bi sites. It is not obvious which of these sites would be doped with Sb, therefore no artificial doping is performed.

**Fig. 2:**
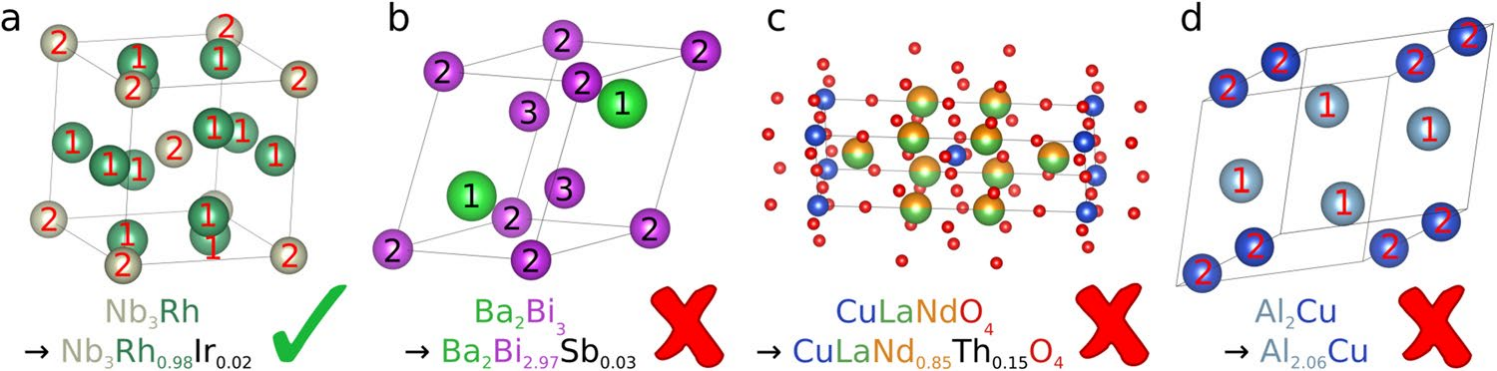
Examples of SuperCon entries and respective candidate crystal structures before artificial doping. The checkmark shows if it is possible to use artificial doping to modify the chemical formula of the crystal structure (top) to fit the chemical formula of the SuperCon entry (bottom). The numbers on the atoms denote each set of crystal sites with the same Wyckoff position. (**a**) shows a crystal with only one Wyckoff position for all Rh sites, (**b**) shows a crystal with two different Wyckoff positions for the Bi sites, (**c**) would generate a crystal structure with three elements on one site, and (**d**) would require an additional crystal site.
  We define equivalent crystal sites as all crystal sites which would have the same probability of being doped with a certain element, i.e. the sites have the same Wyckoff position (they are symmetrically equivalent) or the sites are already doped or partially occupied by the same elements in the same quantities and therefore empirically behave identically under doping. The latter condition is important since a large number of cuprates in the ICSD would otherwise be excluded.
- *The replacement does not lead to crystal sites with more than two elements:* Fig. 2c shows an example of this requirement. Even though the necessary replacement of elements would be unambiguous, we decided to discard such cases, so that each crystal site is doped with at most two different elements.
- *Artificial doping does not add or remove a crystal site:* Artificial doping is supposed to introduce only a minor bias. As such, it is acceptable to slightly modify the occupation numbers quantitatively, but fully removing a crystal site would constitute a severe change in the crystal structure. Furthermore, adding a crystal site is not possible since its position cannot be determined without further analysis. This is illustrated in Fig. 2d. Note that this only applies to full crystal sites. Statistical vacancies are still included as partial occupancies.

If requirements (a–d) are met, the appropriate quantity of the host element is replaced with the guest element at all equivalent crystal sites. In case of statistical vacancies, the guest element that is introduced is nothing, which effectively just decreases the total occupancy of the crystal sites. In the ICSD, each element has its oxidation state given. When doping in a completely new element, its oxidation state is not known. In this case, we simply use the oxidation state of the host element for the new element. Even with artificial doping not all SuperCon entries can be matched with a crystal structure. These entries are discarded.

#### Keep only best matches

The algorithm described above will generally match multiple crystal structures to each SuperCon entry. Therefore we identify the best matches by applying specific criteria: In the case of the Materials Project dataset, we first rank by the energy above hull *E*_hull_ (which is calculated in the Materials Project dataset for each crystal structure) and then by Δ_totrel_. In both cases, lower values are preferred. If more than one crystal structure has the same optimal *E*_hull_ and Δ_totrel_, both are kept in the database. In the case of the ICSD dataset, the ranking criterion is whether the crystal temperature is reported (preferred structures are the ones where the crystal temperature was given). If multiple crystal structures fulfill this criterion, all of them are added to our database. The ranking criteria were determined using hyperparameter optimization (see Supplementary Information S2.1). The final 3DSC databases contain multiple crystal structures matched with the same SuperCon entry. This one-to-many mapping arises because multiple crystal structures might have the exact same rank after sorting. In the case of the 3DSC_MP_, out of 5,759 SuperCon entries, only 14 are matched with each 2 crystal structures, so this is not a dominating issue. However, in the case of the 3DSC_ICSD_, the 9,150 SuperCon entries are matched with 86,490 crystal structures (see statistical analysis in Sec. ‘Statistical data analysis’).

## Data Records

The 3DSC_MP_ and the 3DSC_ICSD_ datasets are publicly available on figshare^30^. For the 3DSC_MP_, the directory ‘cifs/’ contains the cif files of all structures in the 3DSC_MP_. The file ‘3DSC_MP.csv’ contains a table with all 5,759 entries in the 3DSC_MP_. Note that for the 3DSC_ICSD_, only the chemical formula and *T*_c_ of each material as well as the ICSD ID of the original ICSD structure are given in the file ‘3DSC_ICSD_only_IDs.csv’ due to the restrictive ICSD license permissions. In order to generate the full 3DSC_ICSD_ database, an ICSD license is required. Once the structures are downloaded, the matching and adaptation algorithm as described in our GitHub repository can be performed (https://github.com/aimat-lab/3DSC).

The most important entries of the 3DSC_MP_ and the 3DSC_ICSD_ are the chemical formula, the critical temperature in Kelvin, and the path to the CIF file which contains the corresponding three-dimensional crystal structure of each material. Non-superconductors are encoded as having a critical temperature of *T*_c_ = 0 K. Additionally, the 3DSC_MP_ contains additional information from the original Materials Project dataset, e.g. electronic features derived from the original structures (before artificial doping) such as the band gap, the Fermi energy, the energy above hull, or the total magnetization. The electronic and phonon density of states and band structures (if available in the Materials Project) are retrievable using task IDs.

The 3DSC_ICSD_ in its full form (after re-running the matching and adaptation algorithm with structures from the ICSD) has similar entries as the 3DSC_MP_. One difference is that structures from the ICSD do not contain any electronic features such as the band gap or the Fermi energy of the original structures. However, the 3DSC_ICSD_ contains the crystal temperature *T*_cry_ at which the structures were measured, which is missing in the 3DSC_MP_. Since *T*_cry_ was not reported for all of the structures and in doubt assumed to be room temperature (see Sec. ‘Data and dataset cleaning’), an additional binary entry indicates whether *T*_cry_ was given explicitly in the ICSD or not. Both datasets also contain entries that were important for the matching and adaptation algorithm and the analysis in this paper. These entries are important to simplify the reproduction and further work on improving the 3DSC. A more in-depth description of the entries and their exact names in the 3DSC_MP_ and the 3DSC_ICSD_ can be found in the 3DSC repository (https://github.com/aimat-lab/3DSC).

## Technical Validation

### Statistical data analysis

**Fig. 3:**
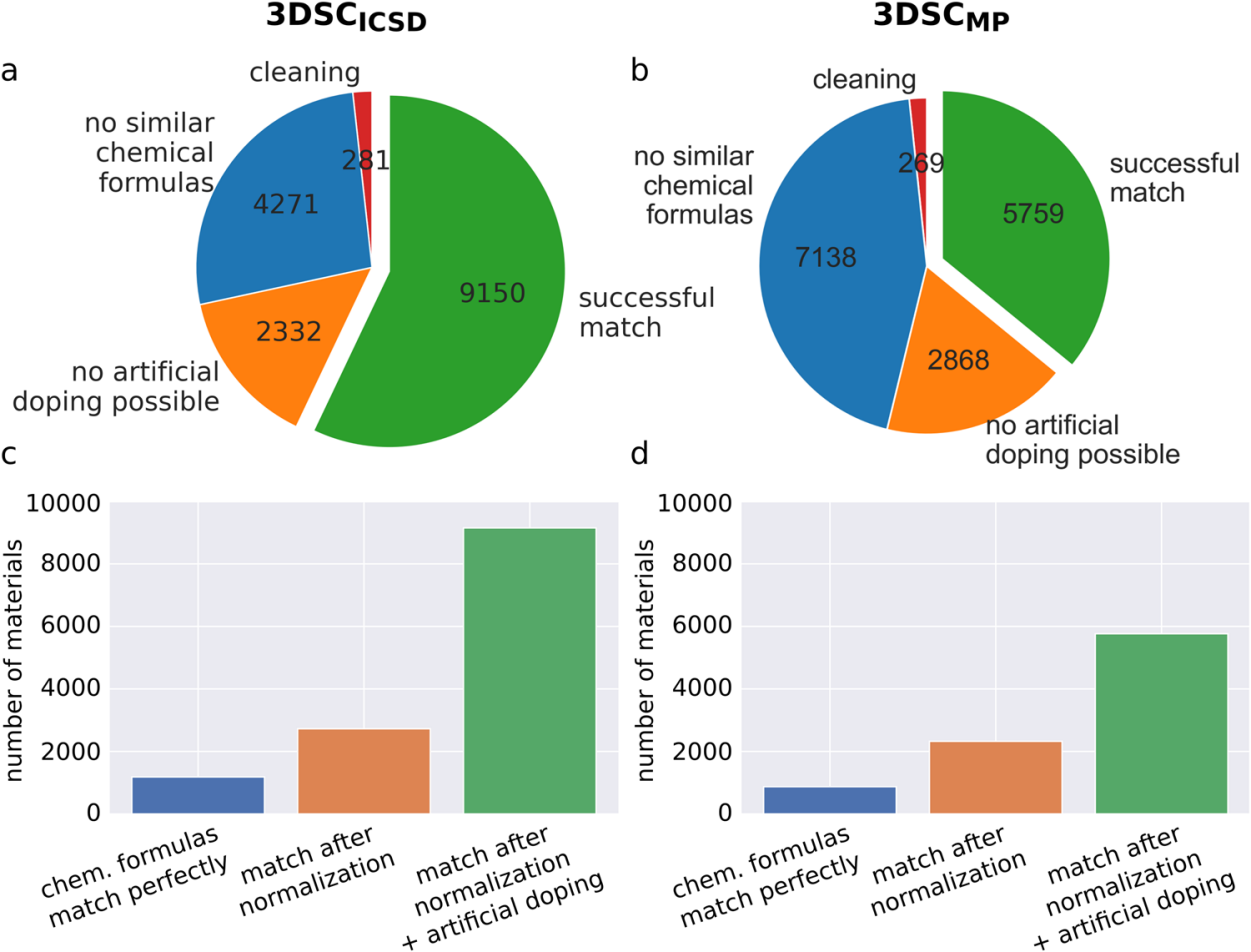
Statistics of the matching algorithm. Panels (**a,b**) show the number of SuperCon entries that are lost in each step of the algorithm for the 3DSC_ICSD_ and the 3DSC_MP_ dataset, respectively. Panels (**c,d**) show the numbers of SuperCon entries in the final dataset that were perfectly matched with crystal structures based on the absolute chemical formula, the normalized chemical formula, and the amount generated using the artificial doping algorithm.

Fig. 3a,b show the cleaning and matching statistics for the 3DSC_ICSD_ and the 3DSC_MP_, respectively. ‘No similar chemical formulas’ means that no crystal structure is close enough to be matched based on the metrics presented in Sec. ‘Matching algorithm of SuperCon entries and 3D crystal structures’. ‘No artificial doping possible’ means that artificial doping can not be performed for one of the other reasons explained in Sec. ‘Matching algorithm of SuperCon entries and 3D crystal structures’. As a result of the matching algorithm, approximately 57% of SuperCon entries can be matched with crystal structures from the ICSD and approximately 36% of SuperCon entries can be matched with structures from the Materials Project. Approximately 93% (5337) of the materials in the 3DSC_MP_ are also in the 3DSC_ICSD_.

The bar plots in Fig. 3c,d show how many material-crystal structure matches are obtained by performing the proposed matching algorithm in contrast to previously reported matching methods which only compare the absolute chemical formulas^7^. Matching normalized chemical formulas as well as performing artificial doping significantly increases the number of matched materials. While normalizing chemical formulas before matching is simple, it doubles the amount of SuperCon entries that can be matched for the 3DSC_ICSD_ and even triples it for the 3DSC_MP_. In addition, artificial doping roughly triples the matched materials for each dataset again. In contrast to standard matching, our proposed matching algorithm leads to a gain of 773% and 660% of matched SuperCon entries for the 3DSC_ICSD_ and the 3DSC_MP_ respectively.

**Fig. 4:**
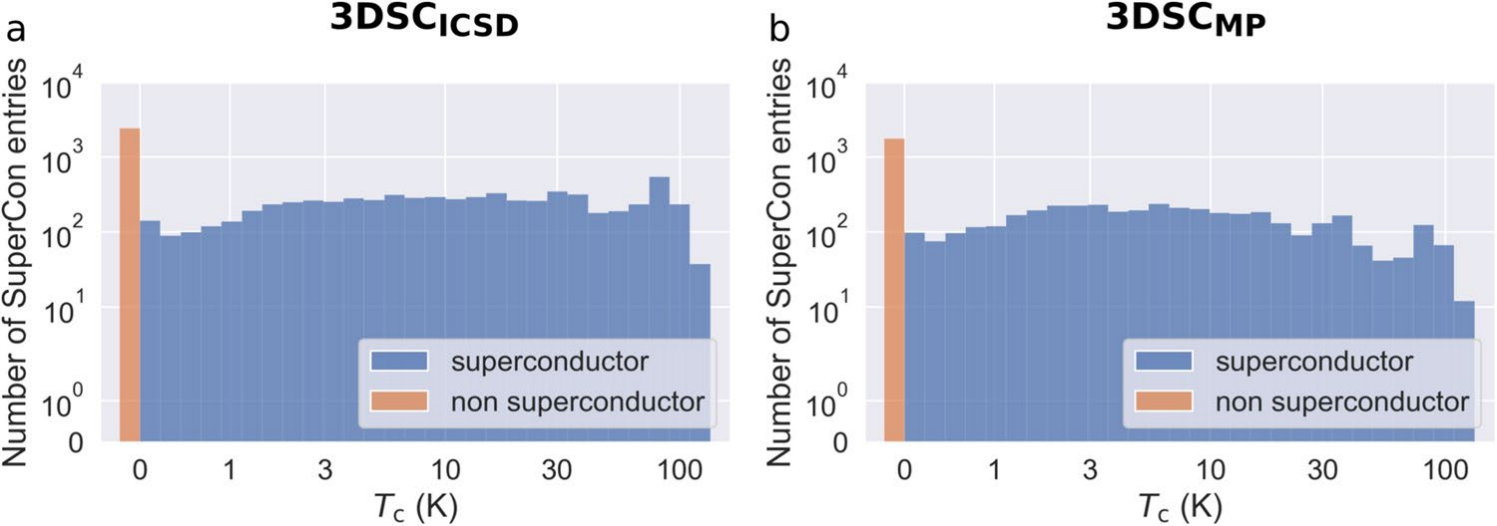
The distribution of SuperCon entries per critical temperature *T*_c_ for the 3DSC_ICSD_ (**a**) and the 3DSC_MP_ (**b**).

When training machine learning models on datasets, an unbalanced distribution of labels can introduce bias, leading to systematic over- or underestimation of the critical temperatures in certain ranges. Fig. 4a,b shows the distribution of *T*_c_ in the datasets for the 3DSC_ICSD_ and the 3DSC_MP_ database, respectively. On a logarithmic scale, the number of superconductors per *T*_c_ is relatively constant. One exception is the relatively high number of superconductors with a critical temperature of approximately *T*_c_ = 90 K, which can be attributed to a widely studied class of superconductors based on YBa_2_Cu_3_O_7_ which has a critical temperature of *T*_c_ = 92 K. Elemental prevalence plots and further statistics of the distribution of *T*_c_, broken down into different groups of superconductors, can be found in the GitHub repository as well.

Finally, it is important to evaluate the statistical influence of having multiple crystal structures per chemical formula in the 3DSC_ICSD_ database. This is an issue mostly for the 3DSC_ICSD_ since the 3DSC_MP_ dataset rarely has multiple structures per SuperCon entry. However, with a different choice of the sorting criteria, this issue would also exist for the 3DSC_MP_ since it is an inbuilt consequence of the matching approach.

**Fig. 5:**
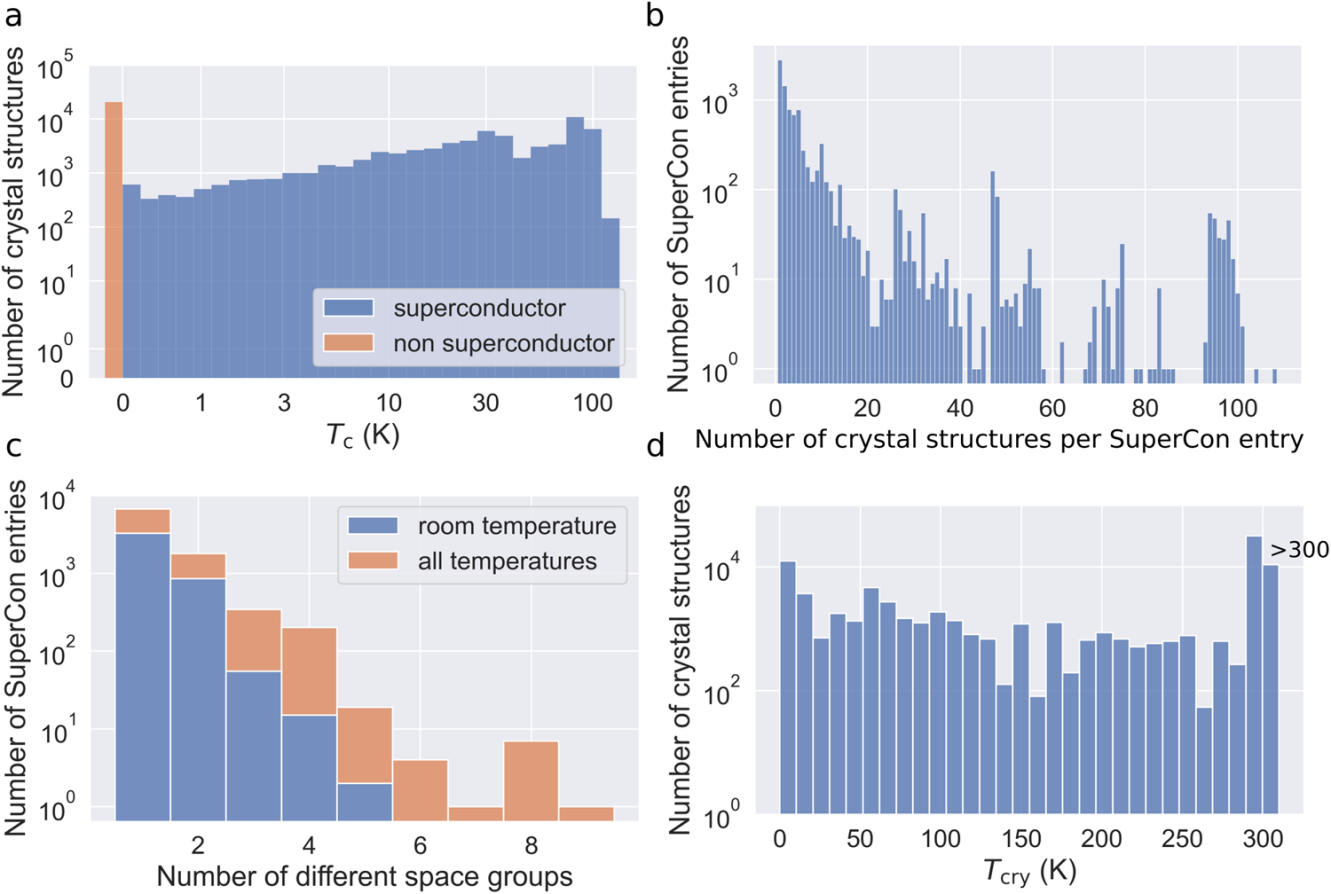
Statistics regarding the mapping of one SuperCon entry to multiple crystal structures in the 3DSC_ICSD_. (**a**) shows a histogram of the number of crystal structures per *T*_c_. (**b**) shows the number of candidate crystal structures generated by the artificial doping algorithm per SuperCon entry. (**c**) shows a histogram of the number of different space groups for each SuperCon entry, both for all crystal temperatures and only for structures at room temperature. (**d**) shows the number of crystal structures at a given crystal temperature *T*_cry_. For a better overview, all structures with a crystal temperature *T*_c_ > 300 K are collected in the last bar. 50% of the crystal structures have a crystal temperature *T*_cry_ < 273 K.

Fig. 5a shows *T*_c_ dependent crystal structure counts in the dataset. For comparison, Fig. 4a shows the distribution of different superconductors instead of crystal structures. We find that the ratio of high-*T*_c_ data points to low-*T*_c_ data points has increased (ignoring the very last bin in the histogram). This shows that high-*T*_c_ superconductors such as cuprates have matched with more crystal structures per material than low-*T*_c_ superconductors. This might pose a potential issue because the effective weight of high-*T*_c_ superconductors to low-*T*_c_ superconductors has shifted from what it was before. To mitigate this issue, we have used a sample weight equal to the inverse of the number of crystal structures per material in all machine learning experiments.

Some materials have matched a lot of crystal structures as shown in Fig. 5b. Note that there is an exponential decrease until approximately 20 crystal structures per SuperCon entry. Most data points beyond this number are artifacts due to series measurements of the same crystal with varying temperatures. Furthermore, some materials have matched crystal structures with a large number of different space groups as shown in Fig. 5c. The color coding shows that many of these different space groups are data points that are measured at room temperature. This potentially poses an issue, because a machine learning model trained on the data will “see” many different crystal structures with different space groups and the same chemical formula, which all have the same critical temperature *T*_c_. This problem is partially mitigated by having many crystal structures measured at low temperatures as shown in Fig. 5d. Note that for a better overview, all of the data points with *T*_c_ > 300 K are collected in the last bar. We assume that the low-temperature crystal structures are helpful in predicting *T*_c_, because they are more likely to be the superconducting structures. However, the issue of having multiple very different crystal structures mapping to the same critical temperature in the 3DSC_ICSD_ has to be kept in mind and will be discussed in Sec. ‘Limitations and perspective’.

### Machine learning results

For all experiments in this section, we have used a gradient boosting (XGB) model with the default hyperparameters of the XGBoost Scikit-Learn API version 1.4.0 (https://xgboost.readthedocs.io/en/release_1.4.0/python/python_api.html#module-xgboost.sklearn). For the cross-validation, we have used *n* randomly repeated 80:20 splits (*n* = 25 for 3DSC_ICSD_, *n* = 100 for 3DSC_MP_). In all our machine learning experiments, following Meredig *et al*.^21^ we accounted for some extrapolation between the train and test set to make the task more realistic: We grouped the materials in the train and test set by their chemical system so that materials with the same chemical system are either all in the train set or all in the test set. The chemical system of a material is defined as the set of all chemical elements which make up the material (incl. dopants), e.g. Ba-Cu-O-Y for YBa_2_Cu_3_O_7_. We also played around with using Random Forests or Neural Networks, but decided to use XGB models without hyperparameter optimization, so that an additional validation set would not be necessary, since this would have been difficult to implement with the large number of repetitions. Even though this initial small trial was based on the test set, due to the large number of repetitions of train/test splits we believe the reported performance to be a representative measure of the dataset.

We computed the Mean Squared Logarithmic Error (MSLE) for each repetition and report the mean as well as the standard error of the mean of the MSLE values. The MSLE metric was also used as the loss function of every single model, due to the distribution of *T*_c_ values in our dataset. Whenever possible, the same train-test splits were used in different experiments. Additionally, each crystal structure was given a sample weight of the inverse of the number of crystal structures for this SuperCon entry, so that the total weight for every SuperCon entry was the same.

Before being passed to the XGB model, the critical temperature *T*_c_ was approximately logarithmically scaled using ${T}_{{\rm{c}}}^{{\prime} }={\rm{arcsinh}}\left({T}_{{\rm{c}}}/2{T}_{{\rm{c}}}^{0}\right)$ with ${T}_{{\rm{c}}}^{0}=1\,{\rm{K}}$. The arcsinh(*x*/2) was chosen because it converges faster to log(*x*) than log(*x* + 1). To represent the chemical formula as a numerical vector we used MAGPIE features^10^. To represent the crystal structures we developed disordered SOAP (DSOAP) features, an extension of SOAP features^37^ for disordered crystal structures, and some symmetry information, as explained in the Supplementary Information S1. Additionally, we concatenated the MAGPIE features of the chemical formula when representing the crystal structure.

#### Importance of structural information

**Fig. 6:**
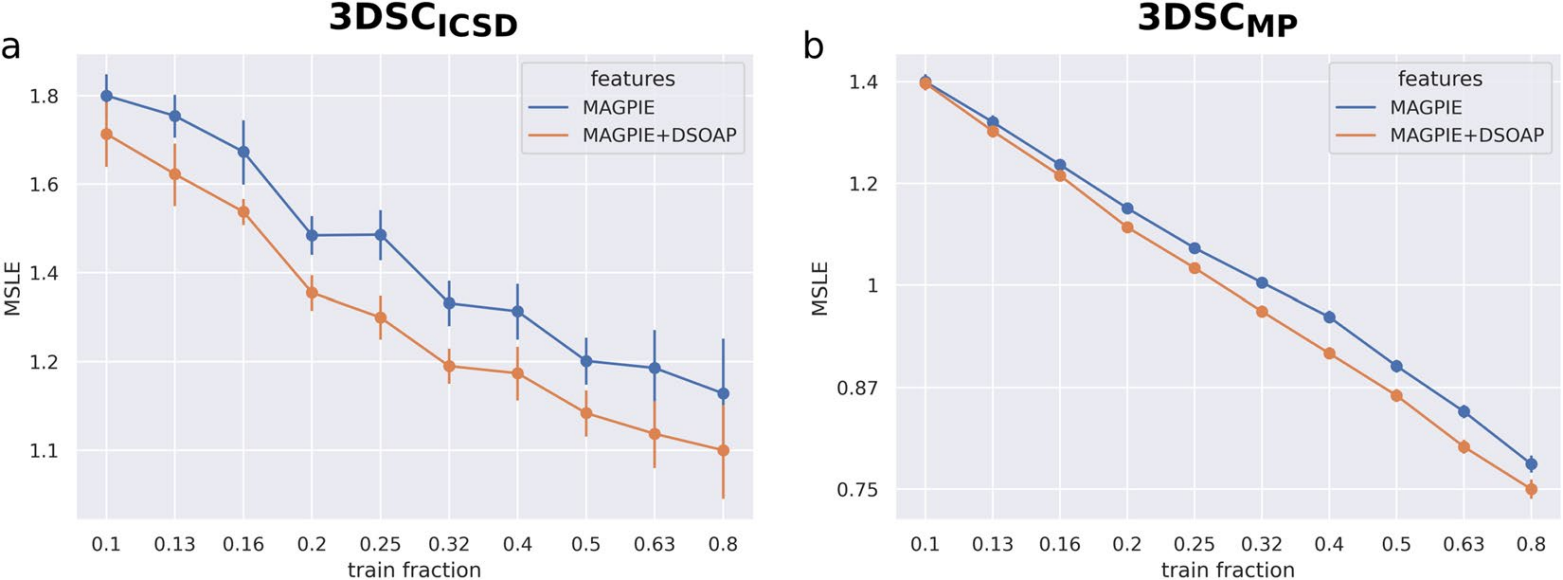
A log-log plot of the learning curve of the 3DSC_ICSD_ (**a**) and the 3DSC_MP_ (**b**) with and without access to structure-aware MAGPIE + DSOAP features. Note the different scales of the MSLE axes in (**a**) and (**b**).

The performance of the XGB models trained with structural information on the 3DSC_ICSD_ and the 3DSC_MP_ dataset is shown in Table 2. Additionally, the performances on both datasets when trained only on the chemical formula are shown as a reference. Fig. 6 shows learning curves in which the performance of the XGB model with and without structural information is plotted for different train set sizes.

**Table 2:** The final results of the XGB models trained on the MAGPIE (“chem. formula”) and the MAGPIE + DSOAP features (“structure”) for the 3DSC_ICSD_ and the 3DSC_MP_. We report the mean and standard error of the MSLEs of 25 and 100 randomly repeated 80:20 splits of the 3DSCICSD and the 3DSCMP respectively.

Dataset	Features	MSLE (test)	MSLE (train)
3DSC_ICSD_	Chem. formula	1.176 ± 0.095	0.155 ± 0.005
	Structure	**1.085 ± 0.073**	0.275 ± 0.008
3DSC_MP_	Chem. formula	0.776 ± 0.010	0.078 ± 0.001
	Structure	**0.748 ± 0.010**	0.135 ± 0.001

For both the 3DSC_ICSD_ and the 3DSC_MP_, training on the structural information improves the prediction of the critical temperature *T*_c_, despite the noise introduced by probably not always matching the correct structure. This is true even in the low-data regime as shown in Fig. 6. As an example, to convey a sense for the MSLE, an MSLE of 0.748 for a superconductor with a true *T*_c_ of 1 K, 10 K and 100 K corresponds to an absolute error of 1.16 K, 6.37 K and 58.47 K respectively. These numbers have been calculated by using the definition of the Squared Logarithmic Error ${\rm{SLE}}\left(y,\widehat{y}\right)={\left(ln(1+y)-ln(1+\widehat{y})\right)}^{2}$ with true target *y* and predicted target $\widehat{y}$. Rearranging and solving for $\widehat{y}$ gives $\widehat{y}=\left(1+y\right)\exp \left(-\sqrt{SLE}\right)-1$. Using the definition of the absolute error ${\rm{AE}}=\left|y-\widehat{y}\right|$ leads to the values stated above. In general, the MSLE is lower for the 3DSC_MP_ than for the 3DSC_ICSD_. We assume that this is due to the fact that the 3DSC_ICSD_ contains more doped materials. In such materials, slight changes in the feature space can correspond to large changes in *T*_c_. Therefore, the 3DSC_ICSD_ might be harder to predict than the 3DSC_MP_.

Furthermore, while the test error of the models with structural information is smaller, the training error is actually larger than when training on the chemical formula. This is a sign that including the structural information leads to less overfitting of the models. These results show that information about the 3D structure of the crystal structure is crucial for the prediction of the critical temperature, particularly for better generalization.

A consequence of the matching algorithm is that the 3DSC_ICSD_ and the 3DSC_MP_ contain fewer materials than the original SuperCon database from Stanev *et al*.^7^. As a reference, we compare our new XGB model trained on the 3DSC database with DSOAP features (see Table 2) to XGB models trained on the full SuperCon data with MAGPIE features. To make the results comparable, we used exactly the same test sets as before. Additionally, to stay within the extrapolation setting, we removed materials with chemical systems which already occur in these test sets. The resulting MSLE on the test set is 1.092 ± 0.028 for the 3DSC_ICSD_ and 0.704 ± 0.006 for the 3DSC_MP_. It is not a surprise that for the 3DSC_MP_, these results are slightly better than when training on the 3DSC_MP_ (MSLE = 0.748), which contains only 36% of the materials in the full SuperCon. In contrast, the fact that models trained with structural information on the 3DSC_ICSD_ perform better (MSLE = 1.085) than when trained on the full SuperCon with twice as much data shows how useful the structural information is. This shows the potential of data-driven approaches, given that enough data is published according to FAIR and AI-ready standards^38,39^.

#### Importance of structural information for different groups of superconductors

The SuperCon dataset is very clustered, with many data points coming from narrow groups of materials. Having access to the 3D crystal structure information might influence different superconductor groups in different ways. In order to test the performance within different groups of superconductors with and without structural information, we have partitioned the 3DSC_ICSD_ and the 3DSC_MP_ dataset into seven different datasets containing only one class of superconductors each, namely cuprates, ferrites, heavy fermion materials, Chevrel phases, oxides and carbon-based materials. This grouping is based on the chemical formula and is done automatically when cleaning the SuperCon dataset in the matching and adaptation algorithm. The group “others” consists of many electron-phonon superconductors, but can also include unconventional superconductors. It collects all materials that do not fall into the other classes and at the same time do not have enough data to generate a new class. If a superconductor was attributed to multiple groups, it was excluded from this analysis. The number of materials in each group and for each dataset is shown in Table 3. All following models are trained and tested on only one group of superconductors each.

**Table 3:** The number of different materials in the 3DSC_ICSD_ and the 3DSC_MP_ for each group of superconductors.

Group	3DSC_ICSD_	3DSC_MP_
Others	4068	3525
Cuprates	3189	874
Ferrites	936	517
Heavy fermions	402	418
Oxides	384	310
Chevrel phases	103	74
Carbon-based	46	30

**Fig. 7:**
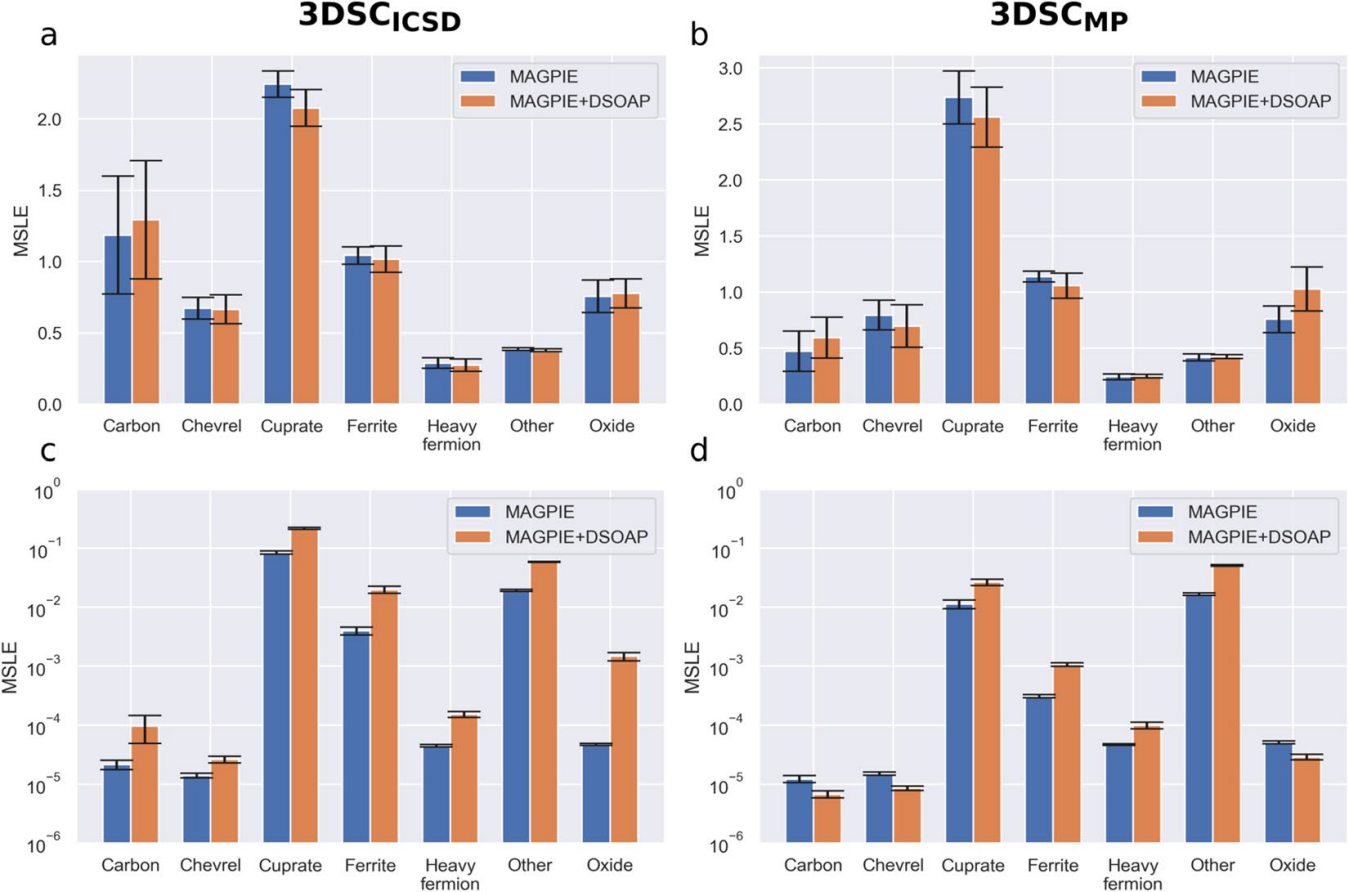
The results of training and testing separately for each superconductor group. For comparison, we show the results with XGB models trained only on MAGPIE features and trained on MAGPIE + DSOAP features. Shown are the mean and standard error of the MSLEs obtained in 5-fold cross-validation grouped by chemical system. The first row shows the performance on the test set for the 3DSC_ICSD_ (**a**) and the 3DSC_MP_ (**b**). The second row shows the performance on the train set for the 3DSC_ICSD_ (**c**) and the 3DSC_MP_ (**d**).

To compare the influence of the structural features for each group independently, we trained XGB models with 5-fold cross-validation grouped by chemical system (see Sec. ‘Machine learning results’) on each group, once only with MAGPIE features, encoding only the chemical formula, and once with MAGPIE + DSOAP features, encoding the crystal structure. The results on the test and train set are shown in Fig. 7. For the test error, the influence of the structurally aware MAGPIE + DSOAP features is not the same across all groups. Overall, the difference in performance between MAGPIE and MAGPIE + DSOAP features is always smaller than the standard error, which makes the difference not statistically significant. However, one can still analyze some trends: The cuprates are the group where the experiments with structural information have the most advantage compared to the runs without structural information. For ferrites, the runs with structural information are a bit better as well. Furthermore, the training error with structural features is higher than the training error without structural features for all groups of the 3DSC_ICSD_ and for the bigger groups in the 3DSC_MP_. This indicates that the structurally aware features are better for generalizing to unseen materials.

What is more interesting is the fact that some of the experiments with structural information have a worse test error than the runs without structural information, in particular for the carbon-based materials and the oxides. We suspect that one reason for this behavior is the way the 3DSC is created, i.e. by matching chemical formulas of materials with the corresponding chemical formulas of crystal structures. One can imagine that such an approach fails for groups such as carbon-based materials, where the normalized chemical formula can look quite similar for very different 3D structures. Technically, it would be most useful for exactly these groups to have the correct structural information. Yet, the way the matching algorithm works, it is also likely to simply match very wrong structures. Another reason might be that at a given dataset size, adding additional features (8000 DSOAP features compared to 145 MAGPIE features) might actually increase overfitting and potentially decrease test set performance. The benefits of additional features will only become statistically significant once a certain training set size is reached (due to steeper learning curves)^40^. Such an overfitting effect can be observed for the smaller groups of the 3DSC_MP_ (carbon-based materials, Chevrel phases, and oxides) where the training error decreases when adding structurally aware features while the test error increases.

For the cuprates, the situation is different: The chemical formula of the structure determines the structure quite well, so the matched structures are likely to be close to the real structures. This might be the reason why the prediction of the *T*_c_ of cuprates works out better with MAGPIE + DSOAP features than only with MAGPIE features.

Overall different groups of superconductors are influenced differently by adding the structural features, even though no clear conclusions can be drawn due to the small dataset sizes. One potential limitation that one should keep in mind is the fact that the matching algorithm might fail just for the groups which would most strongly benefit from structural information, emphasizing again the importance of reporting crystal structures and additional data in a machine-readable and FAIR way.

### Sensitivity analysis

We have performed experiments to investigate the influence of two important parameters in the matching algorithm, the ${\Delta }_{\mathrm{totrel}}^{\max }$ and the normalization of the chemical formulas before matching. The plots and a more detailed discussion of these experiments can be found in the Supplementary Information S2.2 for the normalization of the chemical formulas and in the Supplementary Information S2.3 for the ${\Delta }_{\mathrm{totrel}}^{\max }$. The final ${\Delta }_{\mathrm{totrel}}^{\max }$ used above was chosen to maximize the number of matched materials while minimizing the bias introduced by artificial doping. Our results furthermore show that normalizing the chemical formula before matching and artificial doping is beneficial, indicating that many entries in the SuperCon database do not reflect the exact unit cell. Additionally, we implemented a stricter version of the artificial doping algorithm, where only one single 3DSC_ICSD_ crystal structure is selected for each superconductor, rather than all matching structures. Details can be found in the Supplementary Information S2.4. We found that the mean performance becomes slightly worse, but the shift was not statistically significant.

### Limitations and perspective

The main limitation of the matching algorithm and the 3DSC is that there is no guarantee that a matched crystal structure is the correct superconducting structure for this material. This is particularly obvious for the 3DSC_ICSD_ where there can be up to 9 different space groups for the same SuperCon entry. The 3DSC_ICSD_ tries to mitigate this problem by ‘diluting’ uninteresting structures with more interesting structures. The 3DSC_MP_ tries to counter this problem by using the more stable structures identified by the energy above hull. Ultimately, this problem can only be solved by manually choosing the correct superconducting structure based on expert knowledge or measuring the crystal structure at temperatures close to *T*_c_ in order to find the correct crystal phase.

Until the ranking procedure, the matching and adaptation algorithm is very general and tries to keep as much information as possible. Only the step of selecting which structures are most likely to be the superconducting ones is based on empirical assumptions and thus introduces bias and potentially noise. We, therefore, expect further improvements by adjusting the sorting criteria for ranking crystal structures. Two possible additional sorting criteria are the crystal temperature reported in the ICSD database, and space groups reported for some of the SuperCon entries. The idea behind sorting according to crystal temperature is that structures measured at lower temperatures are more likely to be the superconducting structure. However, this approach might lead to an artificially introduced correlation between *T*_c_ and *T*_cry_. Therefore we decided to not include this criterion in our work. The most likely space group for each material can be identified either by checking the sparse structural information in the original SuperCon database or by checking ICSD structures for keywords regarding superconductivity in the paper titles or abstracts. However, this approach only covers a small fraction of materials.

So far, the 3DSC focuses on adding structural information. A promising addition would be to add electronic information, e.g. from the Materials Project into the 3DSC_MP_. Examples include the electronic structure, the band gap, the total energy and the formation energy. However, the equivalent of artificial doping for these electronic features would at least require additional quantum chemical calculations, if possible at all (e.g. for materials with small doping concentrations).

In this work, we have refrained from merging the 3DSC_MP_ and the 3DSC_ICSD_, since already 93% of the materials in the 3DSC_MP_ are in the 3DSC_ICSD_ and the resulting database could have not been published freely. For future work, it might be interesting to expand the 3DSC_MP_ using other publicly available datasets such as the Crystallography Open Database^28^ (COD) or the Automatic FLOW for Materials Discovery^41^ (AFLOW) database to maximize the number of materials in the 3DSC.

Overall, our work demonstrates the added value of having access to FAIR and machine-readable data on superconducting materials in publicly available databases. We hope that this motivates the scientific community working on superconductivity to publish their research data in public databases, including structural information as well as additional meta-data.

## Usage Notes

We provide the full code used for the dataset generation and the analysis in this paper. In order to simplify reproduction and further work on the 3DSC_MP_, we provide a single python script to generate the 3DSC_MP_, plot most of the statistical plots and generate the learning curves. Note that due to memory constraints, the raw 3DSC_MP_ file on GitHub is missing the DSOAP and MAGPIE features which we used for the machine learning experiments in this paper (see Sec. ‘Machine learning results’). Re-running the matching and adaptation algorithm will also include these feature vectors. Future efforts in training machine learning models on the 3DSC_MP_ can be based on the provided datasets, in particular the readily-available 3DSC_MP_. Future efforts on improving the 3DSC can be based on the provided code.

### Supplementary information

Supplementary Information

## Data Availability

Code and data are available free of charge. The code is provided in our Github repository https://github.com/aimat-lab/3DSC. For reproducibility, the SHA of the final commit for this publication is 2471dd51a298a854cb4f365ebd39e72c7cbf3634. The data is available on figshare^30^.

## References

[1] Yao C, Ma Y (2021). Superconducting materials: Challenges and opportunities for large-scale applications. iScience.

[2] Eley, S., Glatz, A. & Willa, R. Challenges and transformative opportunities in superconductor vortex physics. *Journal of Applied Physics* **130**, 050901, 10.1063/5.0055611. Publisher: American Institute of Physics (2021).

[3] Hor PH (1987). High-pressure study of the new Y-Ba-Cu-O superconducting compound system. Physical Review Letters.

[4] Bardeen J, Cooper LN, Schrieffer JR (1957). Microscopic Theory of Superconductivity. Physical Review.

[5] Saal JE, Oliynyk AO, Meredig B (2020). Machine Learning in Materials Discovery: Confirmed Predictions and Their Underlying Approaches. Annual Review of Materials Research.

[6] *SuperCon*, 10.48505/nims.3837 (2022).

[7] Stanev, V. *et al*. Machine learning modeling of superconducting critical temperature. *npj Computational Materials* **4**, 29, 10.1038/s41524-018-0085-8. ArXiv: 1709.02727 (2018).

[8] Hamidieh K (2018). A data-driven statistical model for predicting the critical temperature of a superconductor. Computational Materials Science.

[9] Chen, T. & Guestrin, C. XGBoost: A Scalable Tree Boosting System. In *Proceedings of the 22nd ACM SIGKDD International Conference on Knowledge Discovery and Data Mining*, KDD ‘16, 785–794, 10.1145/2939672.2939785 (Association for Computing Machinery, New York, NY, USA, 2016).

[10] Ward L, Agrawal A, Choudhary A, Wolverton C (2016). A general-purpose machine learning framework for predicting properties of inorganic materials. npj Computational Materials.

[11] Aketi N, Parachuri S, Dussa HP, Uppara H (2019). Regression of superconducting critical temperature: using a pca-grid search-ada boost regression model. International Journal of Innovative Research in Advanced Engineering.

[12] Matsumoto K, Horide T (2019). An acceleration search method of higher T c superconductors by a machine learning algorithm. Applied Physics Express.

[13] Le TD (2020). Critical Temperature Prediction for a Superconductor: A Variational Bayesian Neural Network Approach. IEEE Transactions on Applied Superconductivity.

[14] Gaikwad, M. & Doke, A. R. Featureless approach for predicting Critical Temperature of Superconductors. In *2020 11th International Conference on Computing, Communication and Networking Technologies (ICCCNT)*, 1–5, 10.1109/ICCCNT49239.2020.9225447 (2020).

[15] Konno T (2021). Deep learning model for finding new superconductors. Physical Review B.

[16] Zeng S (2019). Atom table convolutional neural networks for an accurate prediction of compounds properties. npj Computational Materials.

[17] Li S (2020). Critical Temperature Prediction of Superconductors Based on Atomic Vectors and Deep Learning. Symmetry.

[18] Zhou, Q. *et al*. Atom2Vec: learning atoms for materials discovery. *Proceedings of the National Academy of Sciences* **115**, E6411–E6417, 10.1073/pnas.1801181115. ArXiv: 1807.05617 (2018). 10.1073/pnas.1801181115 PMC6048531 29946023

[19] Dan Y (2020). Computational Prediction of Critical Temperatures of Superconductors Based on Convolutional Gradient Boosting Decision Trees. IEEE Access.

[20] Sizochenko N, Hofmann M (2021). Predictive Modeling of Critical Temperatures in Superconducting Materials. Molecules.

[21] Meredig B (2018). Can machine learning identify the next high-temperature superconductor? Examining extrapolation performance for materials discovery. Molecular Systems Design & Engineering.

[22] Roter, B. & Dordevic, S. V. Predicting new superconductors and their critical temperatures using unsupervised machine learning. *Physica C: Superconductivity and its Applications* **575**, 1353689, 10.1016/j.physc.2020.1353689. ArXiv: 2002.07266 (2020).

[23] Foppiano L (2021). SuperMat: construction of a linked annotated dataset from superconductors-related publications. Science and Technology of Advanced Materials: Methods.

[24] Yamaguchi, K., Asahi, R. & Sasaki, Y. SC-CoMIcs: A Superconductivity Corpus for Materials Informatics. In *Proceedings of the 12th Language Resources and Evaluation Conference*, 6753–6760 (European Language Resources Association, Marseille, France, 2020).

[25] Beltagy, I., Lo, K. & Cohan, A. SciBERT: A Pretrained Language Model for Scientific Text, 10.48550/arXiv.1903.10676. Number: arXiv:1903.10676 arXiv:1903.10676 [cs] (2019).

[26] Court CJ, Cole JM (2020). Magnetic and superconducting phase diagrams and transition temperatures predicted using text mining and machine learning. npj Computational Materials.

[27] Swain MC, Cole JM (2016). ChemDataExtractor: A Toolkit for Automated Extraction of Chemical Information from the Scientific Literature. Journal of Chemical Information and Modeling.

[28] Gražulis S (2009). Crystallography Open Database – an open-access collection of crystal structures. Journal of Applied Crystallography.

[29] *Superconducting Research Database*, https://srd.physics.ucsd.edu/#/ (2018).

[30] Sommer T, Willa R, Schmalian J, Friederich P (2023). Figshare.

[31] Jain A (2013). Commentary: The Materials Project: A materials genome approach to accelerating materials innovation. APL Materials.

[32] *Materials Project*, https://materialsproject.org/ (2011).

[33] Bergerhoff G, Hundt R, Sievers R, Brown ID (1983). The inorganic crystal structure data base. Journal of Chemical Information and Computer Sciences.

[34] Zagorac D, Müller H, Ruehl S, Zagorac J, Rehme S (2019). Recent developments in the Inorganic Crystal Structure Database: theoretical crystal structure data and related features. Journal of Applied Crystallography.

[35] *Inorganic Crystal Structure Database*, https://icsd.products.fiz-karlsruhe.de/ (1978).

[36] Ong SP (2013). Python Materials Genomics (pymatgen): A robust, open-source python library for materials analysis. Computational Materials Science.

[37] Bartók AP, Kondor R, Csányi G (2013). On representing chemical environments. Physical Review B.

[38] Wilkinson, M. D. *et al*. The FAIR Guiding Principles for scientific data management and stewardship. *Scientific Data* **3**, 160018, 10.1038/sdata.2016.18. Number: 1 Publisher: Nature Publishing Group (2016). 10.1038/sdata.2016.18 PMC4792175 26978244

[39] Scheffler M (2022). FAIR data enabling new horizons for materials research. Nature.

[40] von Lilienfeld OA, Burke K (2020). Retrospective on a decade of machine learning for chemical discovery. Nature Communications.

[41] Curtarolo S (2012). AFLOWLIB.ORG: A distributed materials properties repository from high-throughput ab initio calculations. Computational Materials Science.

