# Supplementary material for: 3DSC - a dataset of superconductors including crystal structures
Source: Sci Data. 2023 Nov 21;10:816. doi: 10.1038/s41597-023-02721-y (PMC10663493; doi:10.1038/s41597-023-02721-y)
Supplement: Supplementary file 1 — Supplementary Information [file 41597_2023_2721_MOESM1_ESM.pdf]

Supplementary Information

Contents

S1 Disordered SOAP (DSOAP) features **1**

S2 Additional machine learning experiments **2**

S2.1 Sorting criteria optimization 2

S2.2 Normalized chemical formulas 5

S2.3 The difference of chemical formulas Δ_{totrel} 5

S2.4 Random dropping of crystal structures and importance of symmetry features 7

References **8**

S1 Disordered SOAP (DSOAP) features

To represent the 3D crystal structures for machine learning algorithms, we chose SOAP features[1] which are calculated using the python package Dscribe[2]. Yet, these features only support ordered crystal structures, but not disordered structures with fractional occupancies such as vacancies and doping. Still, such structures frequently appear in the 3DSC. To address this issue we have generalized the SOAP features to 'Disordered SOAP' (DSOAP) features. These DSOAP features are based on the original SOAP features of ordered structures, but they also incorporate information such as doping and vacancies.

We will now explain the implementation of the DSOAP features and give an example. The idea behind DSOAP features is simple: Each disordered structure is understood as consisting of a superposition of ordered structures. The weights in this superposition are given by the occupancies. The SOAP features of the ordered structures can be calculated with the Dscribe library. The DSOAP vector of the disordered structure is then given as a weighted average of the SOAP vectors of the ordered structures, with weights given by the occupancies.

As an example we will discuss the DSOAP features for an (exemplary) crystal with chemical formula $Fe_{0.35}Zn_{0.35}Te_{1.6}Se_{0.4}O_{0.5}$. Assume this crystal structure has four atom sites in its primitive unit cell:

1. $O_{0.5}$ (vacancy)
2. Te (ordered)
3. $Te_{0.6}Se_{0.4}$ (doped)
4. $Zn_{0.35}Fe_{0.35}$ (doped vacancy)

In the following we will explain each step to calculate the DSOAP features of this crystal structure:

1. **Vacancies:** For vacancies, the total occupancy of each atom site is recorded to use it as weight for this atom site. In this example, the recorded vacancy weights for the four sites are (0.5, 1, 1, 0.7). After recording these weights the occupancies of the vacancies are scaled so that afterwards each crystal site has a total occupancy of 1.0. The four atom sites now have the following occupancies:
   1. O (scaled by a factor of $1/0.5$)
   2. Te (unchanged)
   3. $Te_{0.6}Se_{0.4}$ (unchanged)
   4. $Zn_{0.5}Fe_{0.5}$ (scaled by a factor of $1/0.7$)

2. **Doping:** For doped crystal structures, we generate all possible combinations of ordered structures that can arise from the doped elements. These ordered structures serve as proxy structures. Since there are two doped crystal sites, namely $Te_{0.6}Se_{0.4}$ and $Zn_{0.5}Fe_{0.5}$, with two elements each, there are four proxy structures: $FeTe_2O$, FeTeSeO, $ZnTe_2O$, ZnTeSeO.

   Additionally the doping occupancies are recorded as weights for each proxy structure. The weights for all crystal sites of a structure are multiplied to simulate the probabilities. The doping weights for each of the four proxy structures are:
   $FeTe_2O$: $0.5 \cdot 1 \cdot 0.6 \cdot 1 = 0.3$
   FeTeSeO: $0.5 \cdot 1 \cdot 0.4 \cdot 1 = 0.2$
   $ZnTe_2O$: $0.5 \cdot 1 \cdot 0.6 \cdot 1 = 0.3$
   ZnTeSeO: $0.5 \cdot 1 \cdot 0.4 \cdot 1 = 0.2$

   Note that they add up to 1, because we calculated the doping weights for the manipulated structure without vacancies. *Remark:* Because of the combinatorial nature of this algorithm, it can happen that the number of proxy structures becomes extremely high and computationally not tractable when doing this for each crystal site. To alleviate this problem, we calculate the combinations of proxy structures not per crystal site but per symmetrically equivalent set of crystal sites, i.e.

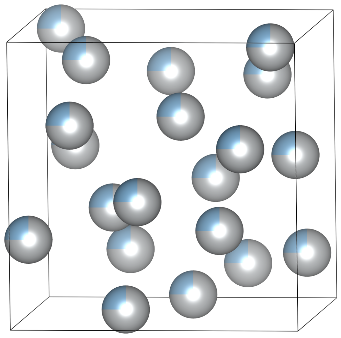

Figure S1. This structure has 20 doped crystal sites in its primitive unit cell, but they all belong to only 2 different Wyckoff positions.

per Wyckoff position. An example for this issue (taken from the ICSD) is shown in Figure S1: The crystal structure of $Ag_{0.75}Al_{0.25}$ has 20 doped crystal sites, but they all belong to only two different sets of Wyckoff positions. Therefore, instead of having to compute 2^{20} different ordered proxy structures we only need to compute 2^2.

3. **SOAP features:** We calculate the SOAP vectors of each crystal site of all proxy structures with the Dscribe library. This is no issue anymore since these structures are completely ordered. Since in our example there are 4 proxy structures with 4 crystal sites each, there are 16 SOAP vectors in total.

4. **Weighted average of crystal sites:** We compute the full SOAP vector of one proxy structure by doing a weighted average over all of its crystal sites. The weights are the recorded vacancy weights, i.e. the original total occupancy of each crystal site. In this example these weights are (0.5, 1, 1, 0.7). The weighted average $\boldsymbol{v}$ of some vectors $\boldsymbol{v}_i$ with weights w_i is defined as

$$\boldsymbol{v}(w_i, \boldsymbol{v}_i) = \frac{\sum_i w_i \boldsymbol{v}_i}{\sum_i w_i} \tag{1}$$

5. **Weighted averages of ordered structures:** We compute the DSOAP vector of the original disordered structure by doing a weighted average over all of the ordered proxy structures. The weights are the recorded doping weights. In this example these weights are (0.3, 0.2, 0.3, 0.2).

Note that the order of the weighted averages doesn't matter since it is just two times a linear combination of vectors after each other:

$$\boldsymbol{c} = \boldsymbol{v}_{\mathrm{dop}}(\boldsymbol{v}_{\mathrm{vac}}(\boldsymbol{s}_{ij}^{\mathrm{proxy}})) = \sum_{i,j} \frac{w_i^{\mathrm{dop}} w_j^{\mathrm{vac}}}{(\sum_k w_k^{\mathrm{dop}}) \cdot (\sum_l w_l^{\mathrm{vac}})} \boldsymbol{s}_{ij}^{\mathrm{proxy}} \tag{2}$$

where $\boldsymbol{c}$ is the DSOAP vector for the final crystal, w_i^{dop} and w_j^{vac} are the weights for the doping and the vacancies and $\boldsymbol{s}_{ij}^{\mathrm{proxy}}$ is the SOAP vector of the jth crystal site of the ith proxy structure.

Note also that if one inputs an ordered crystal structure, the output is reduced to the original SOAP features.

Additionally to the DSOAP features we used symmetry features F_{sym}. This was done simply because we have this symmetry given automatically and we assumed it to be helpful for predicting T_{c} if the symmetries would be encoded explicitly. These symmetry features had 11 entries: The first 7 entries encoded the 7 crystal systems (cubic, hexagonal, monoclinic, orthorhombic, tetragonal, triclinic, trigonal) with the corresponding point group encoded as an integer in these 7 feature vectors. Additionally the bravais-centring (primitive, base-centered, body-centered, face-centered) is one-hot encoded as 4 additional binary features. In all experiments these F_{sym} features are always implicitly appended when using DSOAP features. However, our analysis of the results showed that the symmetry features made our results slightly better in the case of the $3DSC_{\mathrm{ICSD}}$ and slightly worse in the case of the $3DSC_{\mathrm{MP}}$, suggesting that the information added by F_{sym} features is limited (see S2.4).

S2 Additional machine learning experiments

S2.1 Sorting criteria optimization

In this section we will present how we developed the filtering criteria that were used to reduce the number of crystal structures per SuperCon entry. First, we chose 4 different criteria which might have an influence:

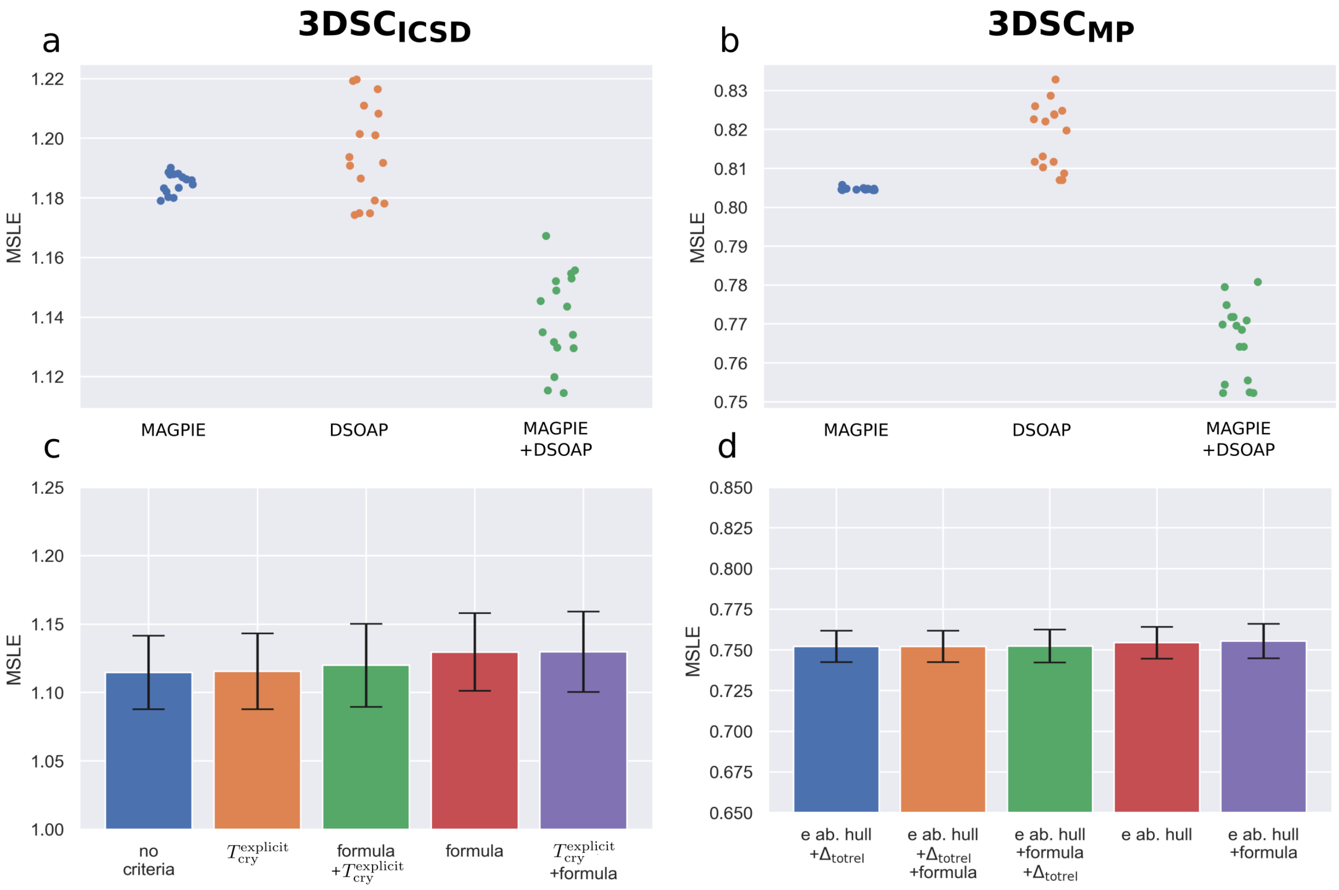

Figure S2. Results of the sorting criteria optimization. (a) and (b) show the MSLE of all runs of the sorting criteria optimization with different sorting criteria for the $3DSC_{ICSD}$ (a) and the $3DSC_{MP}$ (b). Each data point is the mean of 25 repetitions for the $3DSC_{ICSD}$ and 100 repetitions for the $3DSC_{MP}$. (c) and (d) show mean and error of the mean of the top 5 sorting criteria for the $3DSC_{ICSD}$ (c) and the $3DSC_{MP}$ (d) for the run with MAGPIE+DSOAP features sorted by their MSLE.

1. Δ_{totrel}: Entries with a lower Δ_{totrel} are potentially less biased by the artificial doping. This sorting criteria is semi-continuous: Technically it is a continuous number, but due to the integer number of atoms in most chemical formulas, there will still often be multiple crystal structures of one SuperCon entry with the same Δ_{totrel}.

2. Whether or not the chemical formula had to be normalized in order to match: Crystal structures and SuperCon entries potentially match better if their chemical formulas do not have to be normalized to match. This is a binary sorting criteria which means that after sorting by this criteria there will usually still be many crystal structures with the same ranking.

3. Energy above the hull E_{hull} (Materials Project): This criteria applies only to the $3DSC_{MP}$ because only the Materials Project has E_{hull} given. E_{hull} is a property that can be used to predict the stability of the phase of a crystal structure. It is a continuous number and it happens only very rarely that two crystal structures of the same SuperCon entry have the same E_{hull}. Therefore, after sorting and filtering by E_{hull}, there will nearly never be some duplicate crystal structures left.

4. $T_{cry}^{explicit}$(ICSD): This criterion only applies to the $3DSC_{ICSD}$. Not all of the ICSD crystal structures had T_{cry} explicitly given. We assumed that crystal structures which did have T_{cry} explicitly given are more trustworthy. This sorting criteria is categorical.

5. One additional option was to use all data points without any filtering.

The results of the sorting criteria hyperparameter optimization are shown in Figure S2a to d. Figures a and b show for each run the mean of all 100 and 25 cross validation repetitions for the $3DSC_{ICSD}$ and $3DSC_{MP}$ respectively. We plotted all runs of the grid search so that one can be certain that a particularly good result is not only due to overfitting to the test set.

From the plots in Figure S2a and b one can see that the MAGPIE+DSOAP features are consistently better than only MAGPIE or only DSOAP features, for both datasets. This shows that including structural information indeed helps the model in predicting the critical temperature T_c. Interestingly, using only DSOAP features often leads to worse results than using only MAGPIE features. One possible reason is that MAGPIE features include information about chemical closeness of elements, which helps predicting rare elements.

We also tried out to incorporate the electronic features of the $3DSC_{MP}$, but this did not significantly improve the results so we did not include them in the analysis in the main part. The electronic features that we tried were the band gap, energy, energy per atom, formation energy per atom, total magnetization, number of unique magnetic sites and the true total magnetization as recorded in the Materials Project. Note that we did not try to use the Fermi energy E_F because it was not given for all crystal structures in the $3DSC_{MP}$.

It is noticeable that for the $3DSC_{ICSD}$ in Figure S2a the variance of the runs with MAGPIE features is greater than for the $3DSC_{MP}$ in Figure S2b. In theory, each run which uses only MAGPIE features should have exactly the same performance because we controlled that every split has exactly the same SuperCon entries and only the used crystal structures are different between the runs. However, because each SuperCon entry can have a different number of crystal structures, it also appeared a different number of times for the model. In theory this should not matter because we were passing a sample weight with each crystal structure to weigh each SuperCon entry the same. However, we suspect that this randomness is due to the data bagging in the XGB algorithm. Due to the data bagging, different partitions of the data will be used for each decision tree if the dataset is not exactly the same. This effect is much stronger for the $3DSC_{ICSD}$ than for the $3DSC_{MP}$ because the $3DSC_{ICSD}$ has much more crystal structures per SuperCon entry than the $3DSC_{MP}$. One could probably mitigate this issue by fixing that all crystal structures of one SuperCon entry will all be given to the same decision tree.

Figure S2c and d show the top five sorting criteria ordered by their MSLE with mean and error of the mean for the $3DSC_{ICSD}$ and $3DSC_{MP}$ respectively. In the $3DSC_{ICSD}$ (c) simply using all data points indeed is the best option. The second best option with no significant difference in performance is sorting by $T_{cry}^{explicit}$. Because the performance of the two runs has no significant difference we decided to use the latter, because this reduced the number of crystal structures in the $3DSC_{ICSD}$ database from approximately 140,000 to approximately 80,000 and makes consecutive training faster and less memory intensive.

For the $3DSC_{MP}$ (d) it seems that sorting by the energy above the hull E_{hull} is the by far most important criteria. Even though the following option also have other criteria after E_{hull}, these criteria effectively do not matter because E_{hull} is a continuous float value. It is interesting that the algorithm clearly chooses structures with low E_{hull} to be more informative in this dataset. The Materials Project contains a large number of theoretical structures and the Materials Project website marks experimentally confirmed structures, but this parameter is not accessible in the API. The structure with the minimum E_{hull} is usually experimentally confirmed and the most usual structure for this material, so it might be that the algorithm just focused on excluding overly theoretical structures. Finding out the exact role of E_{hull} in this optimization would be an interesting aspect of further research.

In conclusion, we chose the criteria $T_{cry}^{explicit}$as the sorting criteria to use for the $3DSC_{ICSD}$. This matches the 9,150 SuperCon entries in this dataset with 86,490 crystal structures. For the $3DSC_{MP}$ we chose the criteria of sorting first by E_{hull} and then

by Δ_{totrel} as the sorting criteria for this dataset. This matches the 5,759 SuperCon entries in this dataset with 5,773 crystal structures.

S2.2 Normalized chemical formulas

Normalizing the chemical formulas before matching is an important part of the matching algorithm by which a lot of SuperCon entries are matched which otherwise would not be matched. We will now analyze the influence of this normalization step.

Figure S3a and b show the number of crystal structures with a given normalization factor between the chemical formula of the SuperCon entry and the chemical formula of the crystal structure for the $3DSC_{ICSD}$ and the $3DSC_{MP}$ respectively. These histograms show some more insight into matching normalized chemical formulas. One can see that in the histogram there are peaks with particularly many chemical formulas having a certain relative normalization factor. Besides the trivial peak at 1, there is a large peak at 2 and also at 4, 6 and 8. The peak at the 6 is a bit less pronounced. This behavior is consistent both for the ICSD and the Materials Project. These peaks are not symmetrical. That means there are a lot of cases where the chemical formula of the crystal structure is a multiple of 2^n, but not the other way round. This is an indication that authors of SuperCon entries often did not know the exact primitive unit cell and simply wrote down the smallest integer chemical formula.

We also trained an XGB model on the $3DSC_{MP}$ once with all data points and once only with data points where the chemical formula did not have to be normalized. This subset of data points decreases the number of matched SuperCon entries to 58 % (3358 SuperCon entries). The MSLE of this run is shown in Figure S3c. The results show that training on the additional data points with normalized chemical formulas helps significantly in predicting T_c. This shows that doing this normalization is overall beneficial, the additional data that is gained is worth the introduced bias.

We conclude that normalizing the chemical formulas seems to be overall beneficial. However, it is not a surprise that by having more data we get better results. For future studies it would be more interesting to also look at how the extrapolation performance changes for more difficult extrapolation settings, which is the actual benefit of training on crystal structures instead of only chemical formulas.

S2.3 The difference of chemical formulas Δ_{totrel}

Each entry in the $3DSC_{ICSD}$ and the $3DSC_{MP}$ has the parameter Δ_{totrel} which is a measure for how different the chemical formula of the SuperCon entry and the chemical formula of the original crystal structure before artificial doping were. The maximum allowed $\Delta_{\mathrm{totrel}}^{\mathrm{max}}$ is an important cutoff parameter in the matching algorithm. Choosing an appropriate value of $\Delta_{\mathrm{totrel}}^{\mathrm{max}}$ is important because if it is chosen too small, not many SuperCon entries can be matched with crystal structures. In contrast, if it is chosen too big, the introduced bias due to artificial doping will be big because chemical formulas will be matched with crystal structures with which they are actually not related.

Figure S4a and b show a histogram of how many crystal structures in each dataset have a given Δ_{totrel}. In Figure S4a one can see that for the $3DSC_{ICSD}$ the matched crystal structures decrease sharply with increasing Δ_{totrel}. Extrapolating this curve to higher Δ_{totrel} one can conclude that increasing the value of $\Delta_{\mathrm{totrel}}^{\mathrm{max}}$ would not have increased the number of matched crystal structures by much. This is less obvious in Figure S4b for $3DSC_{MP}$ but there is a decreasing trend as well, and the number of data points with $\Delta_{\mathrm{totrel}} \neq 0$ is smaller anyway.

To study the influence of Δ_{totrel} on the prediction accuracy we trained an XGB model on MAGPIE and MAGPIE+DSOAP features to compare the error of the test set dependent on the Δ_{totrel} of each structure. Figure S4c shows the distribution of the Symmetrical Mean Absolute Percentage Error (SMAPE) over the Δ_{totrel} for a model trained on MAGPIE+DSOAP features. For comparison, the same plot is shown in Figure S4d for a model trained only on MAGPIE features.

To analyze the influence of Δ_{totrel} on the prediction accuracy one can look at Figure S4c where the distribution of the Symmetrical Mean Absolute Percentage Error (SMAPE) was plotted over Δ_{totrel} for each data point. We used the SMAPE here because it is a relative error which does not depend on the magnitude of T_c. Thus the plotted distribution is free from the correlation of Δ_{totrel} and T_c. This is important because most cuprates have a Δ_{totrel} between 0 and 0.04, therefore the MAE would have been very high around this Δ_{totrel} without that the model would have actually been worse there, simply because cuprates tend to have a high T_c. Intuitively one would expect that entries with a higher Δ_{totrel} would have a higher error at the prediction, because these structures are only approximated with artificial doping. Such a correlation can not be seen in Figure S4; the SMAPE seems to be independent of the Δ_{totrel}. This shows that we chose $\Delta_{\mathrm{totrel}}^{\mathrm{max}}$ small enough so that artificial doping is a good approximation of the real crystal structures. Additionally Figure S4d shows the same plot, but with an XGB model trained only on MAGPIE features instead of on MAGPIE+DSOAP features. This plot should definitely be independent of Δ_{totrel} because the model was only trained on the chemical formula, which is not influenced by Δ_{totrel}. The distribution looks very similar to the distribution of the model trained on MAGPIE+DSOAP features, which shows again that Δ_{totrel} does not have a big influence on the dataset with structural features. We can also see this from the sorting criteria optimization (see S2.1) because the Δ_{totrel} did not seem to be an important sorting criteria there.

In conclusion, the choice of $\Delta_{\mathrm{totrel}}^{\mathrm{max}}$ was sensible to get a lot of data points, while at the same time not decreasing the prediction accuracy. One could try to increase $\Delta_{\mathrm{totrel}}^{\mathrm{max}}$ until one notices a decrease in the performance, but probably this would

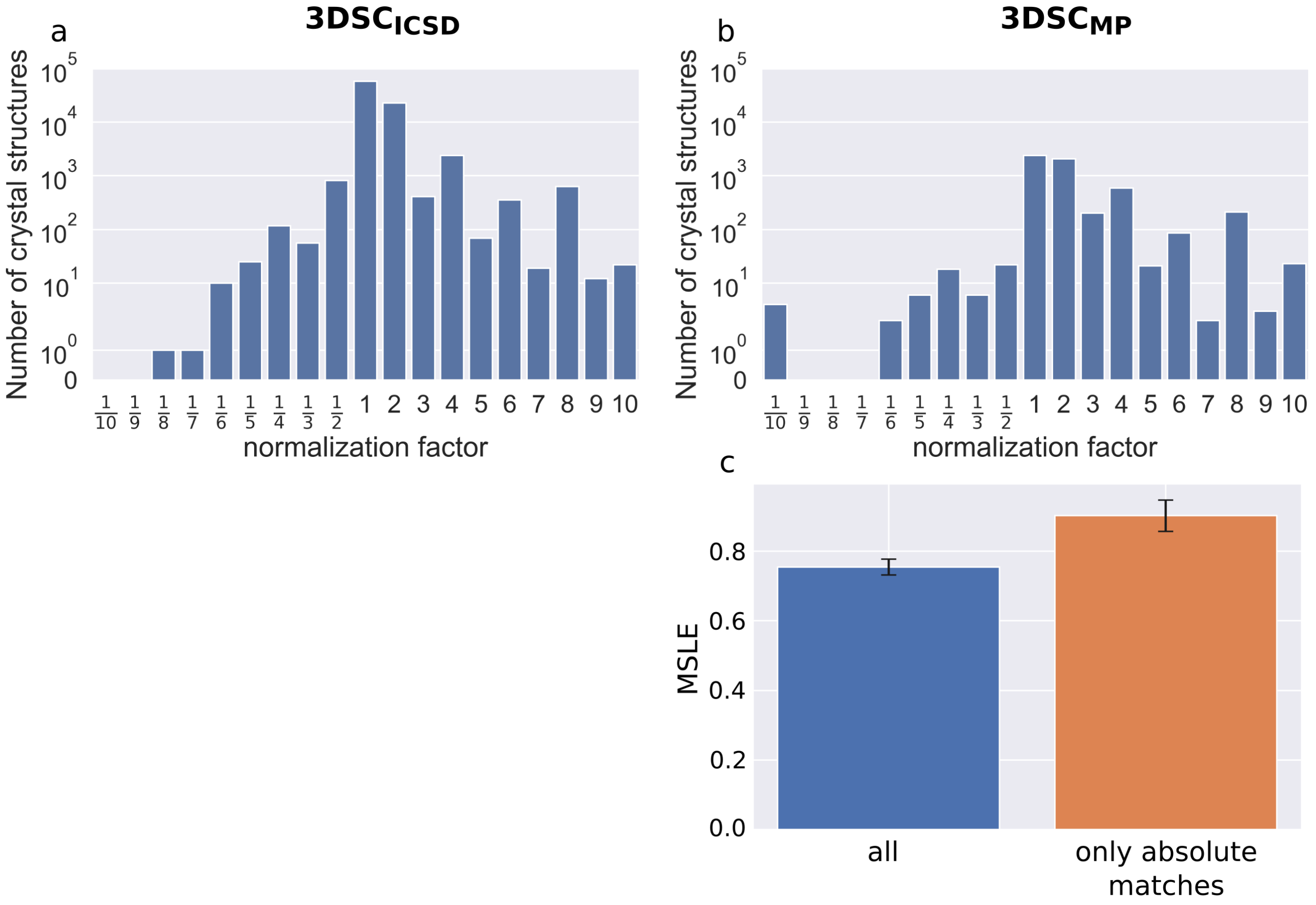

Figure S3. Analysis of the importance of normalizing chemical formulas in the matching algorithm. The first row shows the normalization factor of chemical formulas of the SuperCon entry and the crystal structure for the $3DSC_{ICSD}$ (a) and the $3DSC_{MP}$ (b). For the sake of clarity, the x axis is shown only up to a factor of 10 in both directions. (c) A comparison of training and testing a model on all crystal structures vs only on crystal structures with absolute matches of the chemical formula for the $3DSC_{MP}$. Shown are the mean and the standard error of the mean of 25 repetitions.

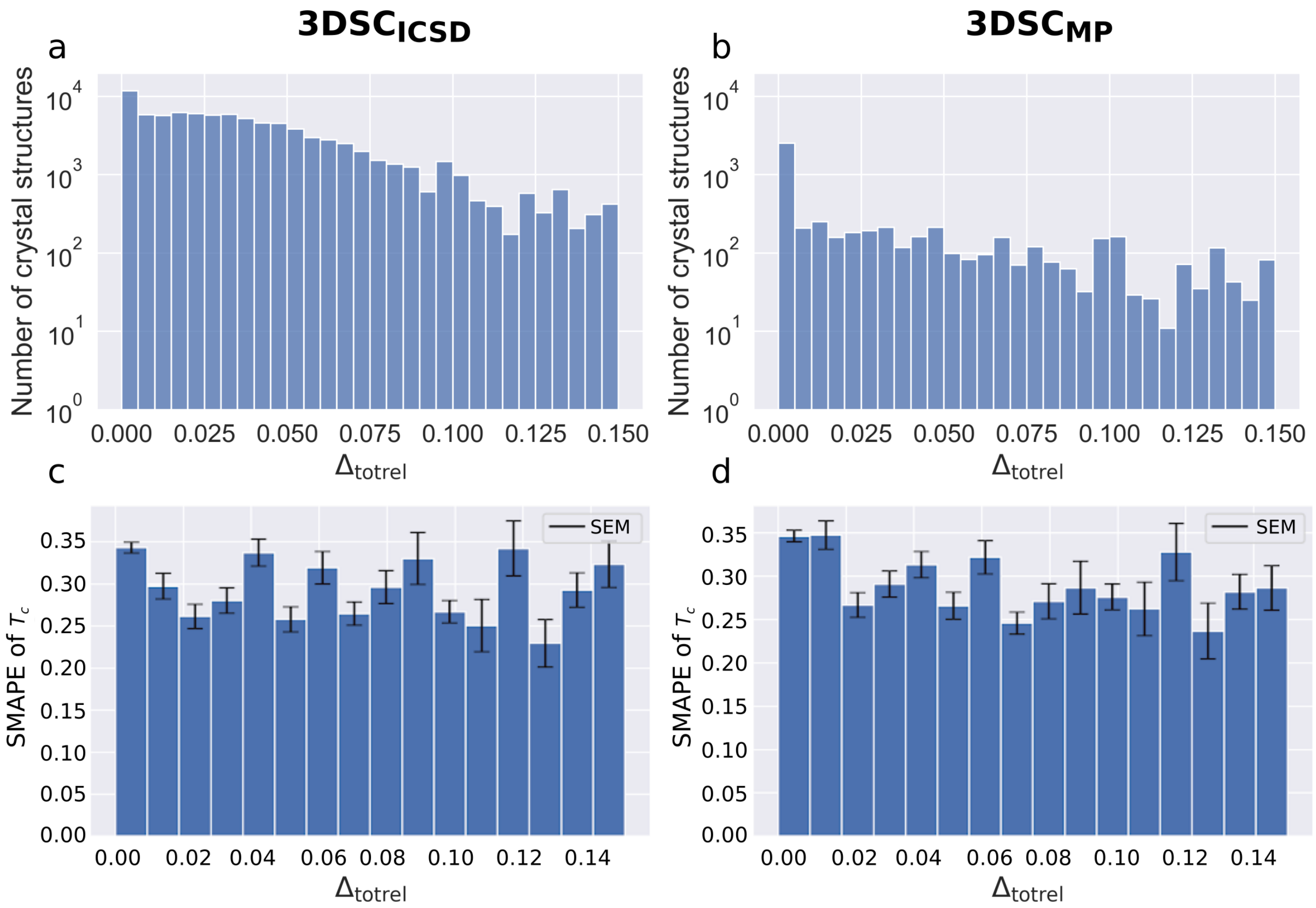

Figure S4. Plots regarding the role of the parameter Δ_{totrel}. The first row shows the number of crystal structures with a given Δ_{totrel} between the chemical formula of the SuperCon entry and the original crystal structure for the 3DSC$_{ICSD}$ (a) and the 3DSC$_{MP}$ (b). The second row shows the Symmetric Mean Absolute Percentage Error (SMAPE) and the standard error of the mean (SEM) of the critical temperature T_c when using MAGPIE+DSOAP features (c) and when using only MAGPIE features (d).

not yield many more data points.

S2.4 Random dropping of crystal structures and importance of symmetry features

In this section we address two independent, little questions. First, we analyze the consequence of randomly dropping all but one crystal structure per SuperCon entry. This would be the simplest version of reducing the number of crystal structures per SuperCon entry to 1. Second, we analyze whether the symmetry features F_{sym} are informative.

We ran experiments with the XGB model as described above. The results in terms of the MSLE are shown in Figure S5a and b. Each plot shows the reference run, the run with randomly dropped crystal structures and the run without symmetry features F_{sym}.

Randomly dropping all but one crystal structure makes the MSLE in case of the 3DSC$_{ICSD}$ slightly worse, but not significantly. For the 3DSC$_{MP}$ this has nearly no effect since there are only very few SuperCon entries with duplicate crystal structures. The fact that randomly dropping crystal structures does not significantly decrease performance is a good sign: On the one hand it allows for faster training without significantly losing performance. It also shows that most of the gain of information that one gets by including the crystal structure is already included if one has just one crystal structure per SuperCon entry. Also, randomly taking just one structure will probably on average be equal to diluting the few non-superconducting structures with superconducting ones, because the dataset is very clustered with many close data points. On the other hand, it is likely that by randomly taking structures there will still be multiple cases where the non-superconducting structure is chosen. Yet that means that there is still room for improvement if one would better choose the crystal structures, which should be studied in more detail in the future.

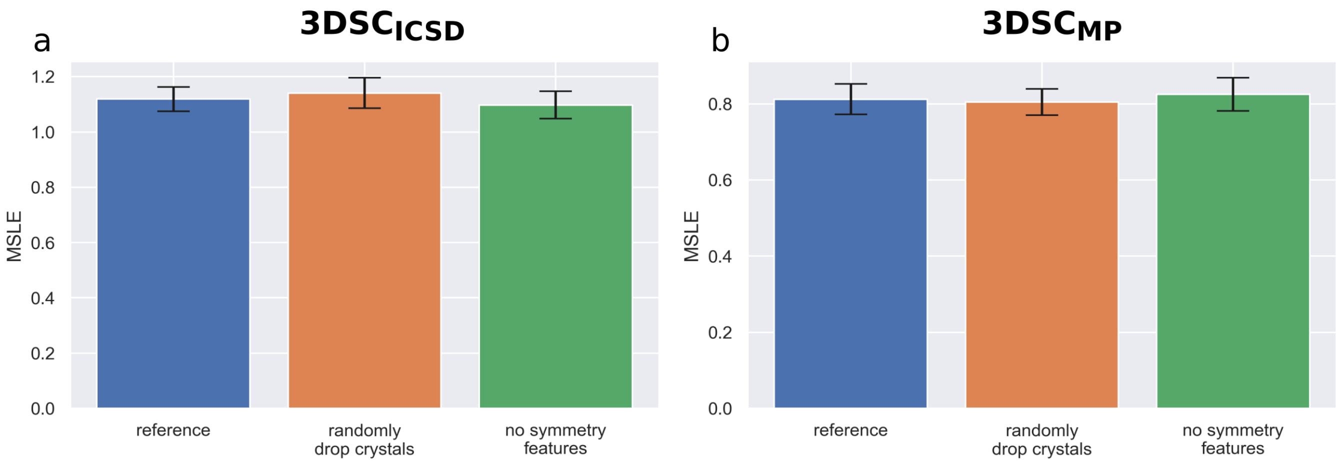

Figure S5. Two independent ablation studies. a and b show a comparison of what happens if one either randomly drops all but one crystal structure for each SuperCon entry or leaves away the symmetry features F_{sym} for the $3DSC_{ICSD}$ (a) and the $3DSC_{MP}$ (b). For comparison a reference run with all crystal structures and including symmetry features F_{sym} is shown.

Training without symmetry features F_{sym} seems to make the training for the $3DSC_{ICSD}$ slightly better and for the $3DSC_{MP}$ slightly worse, but in both cases the changes are not statistically significant.

In conclusion, randomly dropping duplicate crystal structures does not significantly change the performance. The symmetry features on the other hand are not useful for the models with DSOAP features.

References

1. Bartók, A. P., Kondor, R. & Csányi, G. On representing chemical environments. *Phys. Rev. B* **87**, 184115, 10.1103/PhysRevB.87.184115 (2013).
2. Himanen, L. *et al.* DScribe: Library of descriptors for machine learning in materials science. *Comput. Phys. Commun.* **247**, 106949, 10.1016/j.cpc.2019.106949 (2020).
